# Gastroesophageal reflux disease and the risk of respiratory diseases: a Mendelian randomization study

**DOI:** 10.1186/s12967-023-04786-0

**Published:** 2024-01-16

**Authors:** Rui Dong, Qianqian Zhang, Hongxing Peng

**Affiliations:** Department of Respiratory Medicine, Liyuan Hospital, Tongji Medical College, Huazhong University of Science and Technology, Wuhan, Hubei China

**Keywords:** Gastroesophageal reflux disease, Respiratory diseases, Mendelian randomization (MR), Genome-wide association studies

## Abstract

**Background:**

Observational studies have suggested a suspected association between gastroesophageal reflux disease (GERD) and respiratory diseases, but the causality remains equivocal. The goal of this study was to evaluate the causal role of GERD in respiratory diseases by employing Mendelian randomization (MR) studies.

**Methods:**

We conducted Mendelian randomization analysis based on summary data of genome-wide association studies (GWASs) and three MR statistical techniques (inverse variance weighted, weighted median and MR-Egger) were employed to assess the probable causal relationship between GERD and the risk of respiratory diseases. Sensitivity analysis was also carried out to ensure more trustworthy results, which involves examining the heterogeneity, pleiotropy and leave-one-SNP-out method. We also identified 33 relevant genes and explored their distribution in 26 normal tissues.

**Results:**

In the analysis, for every unit increase in developing GERD, the odds ratio for developing COPD, bronchitis, pneumonia, lung cancer and pulmonary embolism rose by 72% (OR_IVW_ = 1.72, 95% CI 1.50; 1.99), 19% (OR_IVW_ = 1.19, 95% CI 1.11; 1.28), 16% (OR_IVW_ = 1.16, 95% CI 1.07; 1.26), 0. 3% (OR_IVW_ = 1.003, 95% CI 1.0012; 1.0043) and 33% (OR_IVW_ = 1.33, 95% CI 1.12; 1.58), respectively, in comparison with non-GERD cases. In addition, neither heterogeneity nor pleiotropy was found in the study. This study also found that gene expression was higher in the central nervous system and brain tissue than in other normal tissues.

**Conclusions:**

This study provided evidence that people who developed GERD had a higher risk of developing COPD, bronchitis, pneumonia, lung cancer and pulmonary embolism. Our research suggests physicians to give effective treatments for GERD on respiratory diseases. By exploring the gene expression, our study may also help to reveal the role played by the central nervous system and brain tissue in developing respiratory diseases caused by GERD.

**Supplementary Information:**

The online version contains supplementary material available at 10.1186/s12967-023-04786-0.

## Introduction

Gastroesophageal reflux disease (GERD) is a chronic digestive disease in which the liquid content of the stomach refluxes into the esophagus [1]. The global prevalence of GERD is high and increasing [2]. In addition to typical regurgitation symptoms, GERD is associated with dysphagia, laryngitis, heartburn, chronic cough, and may develop into esophageal adenocarcinoma [3]. Previous studies indicate the interaction between GERD and respiratory diseases (e.g., chronic obstructive pulmonary disease, asthma, isolated chronic cough, idiopathic pulmonary fibrosis, and cystic fibrosis); [4] however, the causality remains unclear.

Respiratory diseases are closely linked to GERD, and they often present in tandem. A study over varying durations of follow-up (12–18 months) found that GERD is related to a higher chance of experiencing acute exacerbations of chronic obstructive pulmonary disease (risk ratio 7.57 [95% CI 3.84–14.94]) [5]. Patients with persistent respiratory symptoms are highly suspected of having microaspiration and would undertake typical therapy for GERD to address their underlying respiratory problems [6]. Johnson et al. [7] found that patients with typical symptoms of GERD also frequently have respiratory symptoms. In their series of patients who received open Nissen fundoplication for typical symptoms of GERD, 76% of those patients who also had respiratory symptoms experienced relief of those symptoms. However, present studies have drawbacks such as unmeasured confounding, varied interpretation of findings and inadequate statistical robustness due to the small population size. Using Mendelian randomization can fill this gap.

Mendelian randomization (MR) is a form of instrumental variable analysis using genetic variants to evaluate the causal effect of a related exposure on an outcome [8, 9]. Two-sample MR is an extended method of MR using summary statistics from genome-wide association studies (GWAS). This study aims to explore the effect of GERD on COPD, bronchitis, pneumonia, lung cancer and pulmonary embolism in the framework of two-sample MR.

## Methods

### Study design

We performed an MR analysis to explore the causal relationship of GERD with respiratory diseases (COPD, bronchitis, pneumonia, lung cancer and pulmonary embolism) based on GWAS data from a large population. The IV model should meet specific assumptions, including the following [10]: the instrument is highly linked with exposure; the instrument is unrelated to the confounding factors affecting the outcome; and the instrument affects the outcome only through exposure. SNPs strongly associated with GERD were selected as instrumental variables to verify the hypothesis. This study adhered to the genetic principle that alleles are transferred randomly from parents to offspring and that SNPs are unaffected by potential confounding variables such as environmental and socioeconomic status. In addition, we tested for pleiotropic effects which involved determining whether instrumental variables had an influence on outcomes through pathways other than exposure.

### Data source

Summary-level data on the associations of exposure-related SNPs with GERD were derived from Integrative Epidemiology Unit (IEU) public availability (https://gwas.mrcieu.ac.uk/). A total of 129,080 GERD patients and 473,524 controls of European ancestry from the recent GWAS were analyzed [11]. Summary statistics of COPD, bronchitis, pneumonia, lung cancer and pulmonary embolism were obtained from the FinnGen and UK Biobank public availability (Table 1). To reduce the risk of demographic stratification bias, all data were only derived from populations of European ancestry.

**Table 1:** Data source of GERD and respiratory diseases

Phenotype	Description of phenotype	Total sample size	Case; control sample sizes	Cohorts	PMID
Gastroesophageal reflux disease(GERD)	Gastroesophageal reflux disease	602,604	129,080; 473,524	NA	34,187,846
Chronic obstructive pulmonary disease (COPD)	Other chronic obstructive pulmonary disease	218,792	7010; 211,782	FinnGen	NA
Bronchitis	Bronchitis	218,792	27,361; 191,431	FinnGen	NA
Pneumonia	Pneumonia, organism unsepcified	212,258	23,390; 188,868	FinnGen	NA
Lung cancer	Lung cancer	374,687	2671; 372,016	UK Biobank	NA
Pulmonary embolism	finn-b-I9_PULMEMB	218,413	4185; 214,228	FinnGen	NA

Data source of GERD, COPD, bronchitis, pneumonia, lung cancer and pulmonary embolism

### Genetic variant selection criteria

After setting the linkage disequilibrium clumping cutoff to r2 < 0.001 and the genome-wide significance threshold to P = 5 × 10^−8^, we calculated the F-statistics to quantify the strength of genetic variants and subsequently eliminated SNPs with F-statistics less than 10. We then used effect allele frequencies to harmonize the corresponding exposure and outcome datasets. The genetic variants used in the analyses investigating the causal impact of genetically predicted COPD and respiratory diseases are shown (Additional file 1: Table S1). The allele frequencies of SNPs in an average European population are shown in Additional file 1: Table S2. The data were acquired from ALFA Allele Frequency in dbSNP [Home—SNP—NCBI (https://www.nih.gov/)].

### Statistical analysis

The random-effects inverse-variance weighted (IVW) method was conducted for the main study with a complementary analysis using the weighted median and MR-Egger approaches. Meta-analyzing SNP-specific Wald ratio estimates—that is, the beta coefficient for the SNP’s effect on the outcome divided by the beta coefficient for the SNP’s effect on the exposure—with a random-effects or fixed-effects inverse variance method that weights each ratio by its standard error was used to calculate the causal estimates. The weighted median method calculates a weighted median of estimates between SNPs.

In this work, heterogeneity in the analysis was evaluated using the P value of the Cochran’s Q test, and heterogeneity in the causal analysis was regarded to be absent when the Cochran Q-derived p ≥ 0.05. A funnel plot was also applied to detect heterogeneity. In addition, we applied MR-PRESSO to assess the pleiotropy of the model and thus eliminated SNPs that caused bias. By employing MR-Egger regression analysis, the bias caused by genetic pleiotropy may be evaluated, and the magnitude of the pleiotropy could be calculated using the regression intercept [12]. The leave-one-SNP-out method was also used to calculate the combined effect of each remaining SNP.

### Tissue-level SNP heritability enrichment

In this study, we identified genes by searching key SNPs in dbSNP (https://www.ncbi.nlm.nih.gov/snp/). We then performed data mining in Metabolic gEne RApid Visualizer database (MERAV, http://merav.wi.mit.edu) to explore the distribution of 33 genes in 26 normal tissues [13].

## Results

**Fig. 1:**
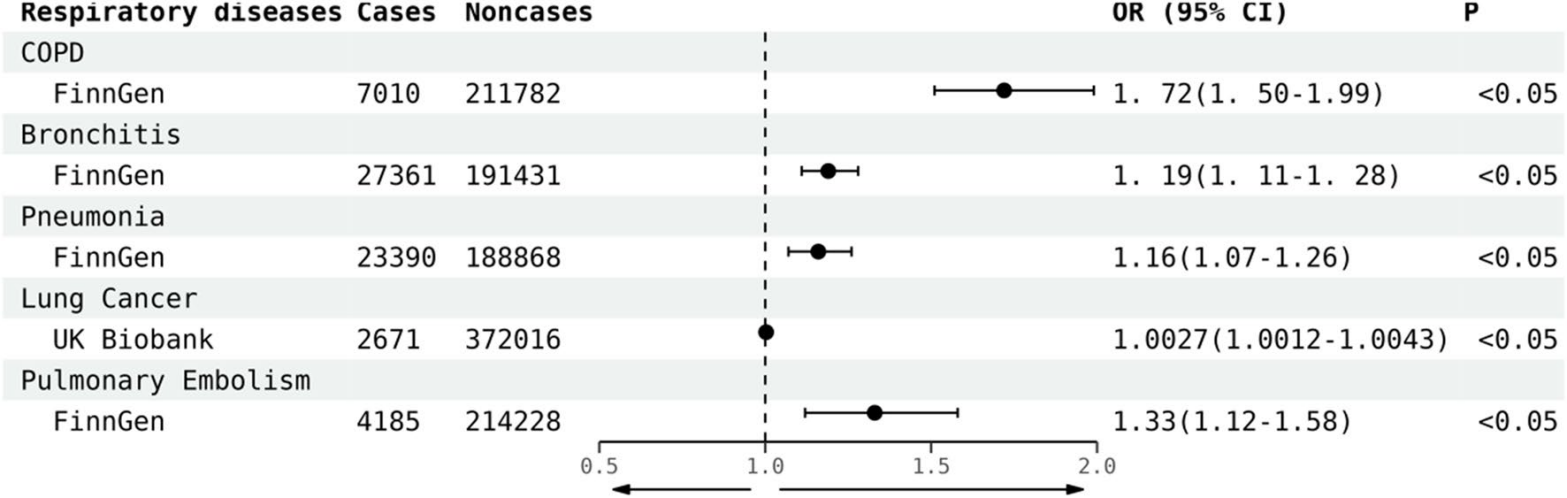
Odds ratios (ORs), 95% confidence intervals and P value for the effect of every unit increase in developing GERD on COPD, bronchitis, pneumonia, lung cancer and pulmonary embolism based on the IVW method

In the analysis, for every unit increase in developing GERD, the odds ratio for developing COPD, bronchitis, pneumonia, lung cancer and pulmonary embolism rose by 72% (OR_IVW_ = 1.72, 95% CI 1.50; 1.99), 19% (OR_IVW_ = 1.19, 95% CI 1.11; 1.28), 16% (OR_IVW_ = 1.16, 95% CI 1.07; 1.26), 0. 3% (OR_IVW_ = 1.003, 95% CI 1.0012; 1.0043) and 33% (OR_IVW_ = 1.33, 95% CI 1.12; 1.58), respectively, in comparison with non-GERD cases (Fig. 1). In addition, we also applied weighted median and MR-Egger, which exhibited a consistent direction of the IVW estimates (Table 2).

**Table 2:** MR estimates for GERD on COPD, bronchitis, pneumonia, lung cancer and pulmonary embolism

Outcome	IVW		WM		MR-Egger	
	OR(95%)	P value	OR(95%)	P value	OR(95%)	P value
COPD	1. 72(1. 50–1.99)	3.53e−14	1.74(1.41–2.14)	2.43e−07	0.80(0.35–1.84)	0.60
Bronchitis	1. 19(1. 11–1. 28)	3.47e−6	1.12(1.01–1.24)	0.029	0.93(0.60–1. 42)	0.72
Pneumonia	1.16(1.07–1.26)	0.00019	1.15(1.03–1.29)	0.015	1.24(0. 87–1.97)	0.37
Lung cancer	1.0027(1.0012–1.0043)	0.00058	1.0036(1.0014–1.0057)	0.00096	1.004(0.996–1.013)	0.34
Pulmonary embolism	1.33(1.12–1.58)	0.0013	1.32(1.02–1.70)	0.034	0.80(0.29–2.22)	0.67

The odds ratio and P value for developing COPD, bronchitis, pneumonia, lung cancer and pulmonary embolism based on IVW, weighted median and MR-Egger results are shown for every unit increase in developing GERD

In total, 76 index SNPs were selected to genetically predict COPD, 76 index SNPs were selected to genetically predict bronchitis, 76 index SNPs were selected to genetically predict pneumonia, 65 index SNPs were selected to genetically predict lung cancer and 76 index SNPs were selected to genetically predict pulmonary embolism. The F-statistics of these SNPs were all larger than 10, suggesting that they are strong instruments.

In the sensitivity analysis, we performed MR-Egger regression and found limited evidence of horizontal pleiotropy in COPD, bronchitis, pneumonia, lung cancer and pulmonary embolism (p = 0.50, 0.94, 0.89, 0.73 and 0.57, respectively). Additionally, no outlier SNPs was identified by employing MR-PRESSO, indicating very weak horizontal pleiotropy. To assess heterogeneity for COPD, bronchitis, pneumonia, lung cancer and pulmonary embolism (p = 0.07, p = 0.25, p = 0.78, p = 0.37 and p = 0.32, respectively), Cochrane’s Q test was applied and all P values were > 0.05. Scatter plots, funnel plots and leave-one-SNP-out analyses are shown in the Additional file 1: Figs. S1–S15.

The MERAV database analysis revealed that relevant gene expression was higher in the central nervous system and brain tissue (Additional file 2: Fig. S16 and Additional file 3: Table S3).

## Discussion

To the best of our knowledge, this is the first large-scale MR study showing evidence that GERD increases the risk for COPD, bronchitis, pneumonia, lung cancer and pulmonary embolism.

In previous studies, the co-occurrence between GERD and COPD patients was reported. When compared with COPD patients who were either asymptomatic or had GERD symptoms less than once a week, those who had both COPD and GERD symptoms at least once a week were more likely to experience more COPD exacerbations [14]. In a cohort study, 1210 COPD patients with GERD and 2420 without GERD were matched to explore the difference in the incidence of ICU admittance and machine ventilation. COPD patients with GERD were more likely to require mechanical ventilation and admission to an intensive care unit based on a retrospective study involving 1210 patients with GERD symptoms [15]. In an observational study, patients with COPD were allocated at random to receive conventional treatments (control group) or conventional treatments with PPIs, and both groups were followed for 12 months. In comparison to the control group, the PPI group had considerably fewer exacerbations per person per year [16]. However, the causal relationship between GERD and COPD is still not clear; thus, an MR analysis focusing on this causality is indispensable.

In a cross-sectional study, to assess how GERD could affect the symptoms of bronchitis, the Frequency Scale for Symptoms of GERD questionnaire was administered to the recruited patients. The presence of GERD causes an increase in sputum symptoms and is associated with an increase in bronchitis symptoms [17]. According to a cohort study, GERD was associated with bronchitis symptoms and exacerbations of respiratory symptoms in a general population sample. GERD subjects had a higher prevalence of bronchitis symptoms than controls (60 percent vs. 26 percent, p < 0.01, respectively) [18]. In another observational study, after receiving cisapride (0.3 mg/kg t.i.d.) for a month, 12 out of 13 children no longer had any nighttime cough [19]. A study focused on future directions in the clinical management of cough emphasized the importance of methods to reliably diagnose and treat bronchitis due to GERD [20]. However, whether bronchitis is caused by GERD is still uncertain, and we need to conduct an MR analysis to explore the causality.

Some researchers have discovered a correlation between GERD and aspiration pneumonia. Intubated, mechanically ventilated patients randomly received cisapride (10 mg, via nasogastric tube) one day and a placebo the other. The cumulative bronchial secretion radioactivity obtained when patients received cisapride was significantly lower than when patients received a placebo: 7540 ± 5330 and 21,965 ± 16,080 cpm, respectively (P < 0.05). In intubated and mechanically ventilated patients, cisapride reduces the quantity of aspirated stomach contents and helps prevent ventilator-associated pneumonia [21]. Despite the fact that associations between GERD and hospital-acquired pneumonias have been demonstrated, community-acquired pneumonias, the major cause of hospitalization and death, did not have conclusive data [22, 23].

Vereczkei et al. noted that patients with non-small cell lung cancer (NSCLC) have significantly higher rates of GERD than the general population [24]. According to a population-based cohort study performed in Taiwan that involved 42,555 people, GERD patients have a higher prevalence of lung cancer than healthy controls [25]. In another multinational cohort study, the researchers found that patients who had anti-reflux surgery had a lower risk of developing lung cancer. The standardized incidence ratios (SIRs) were significantly decreased for small cell carcinoma (SIR = 0.57, 95% CI 0.41–0.77) and squamous-cell carcinoma (SIR = 0.75, 95% CI 0.60–0.92), but not for adenocarcinoma (SIR = 0.90, 95% CI 0.76–1.06) [26]. However, a causal relationship between GERD and other types of cancer has not been verified.

There is no evidence to support that GERD is associated with pulmonary embolism. Therefore, it is crucial to explore the relationship between GERD and pulmonary embolism.

Overall, these results from observational studies collectively indicate that GERD may increase the risk of developing respiratory diseases. However, present studies have drawbacks such as unmeasured confounding, varied interpretation of findings and inadequate statistical robustness due to the small population size. MR analysis is less prone to bias and inverse causation and may enable us to better comprehend the causal relationship between GERD and respiratory diseases.

Several mechanisms may explain these associations between GERD and respiratory diseases. Firstly, it can be explained by the mechanism of neural reflexes, including the reflexes limited to the lower esophageal sphincter and the reflexes involves in the central nervous system. Evidence has shown that GERD might trigger chronic cough by stimulating an esophageal–bronchial cough reflex. Afferent and efferent vagus nerves reflexively carry the neurological impulse to and from the cough center after stimulating a cough sensory nerve ending in the esophagus [27]. Additionally, our research also demonstrated that the expression of selected genes was higher in the central nervous system and brain tissue than in other normal tissues. Previous studies also proved that the pathways of some esophageal and airway sensory nerves terminate in the same regions of the central nervous system [28]. Secondly, except for the mechanism of neural reflexes, respiratory diseases can also result from GERD directly from the gastric contents, which can irritate the upper airways and cause lung disease if aspirated [29]. Distal esophageal acid causes airway irritation and inflammation, which releases bronchoconstrictors. The autonomic innervation between the esophagus and the tracheobronchial tree innervates bronchoconstriction [28]. It is crucial to elucidate the mechanisms underlying GERD raising the risk of respiratory diseases. To assist in the formulation of pertinent clinical recommendations, future studies should confirm the findings and investigate potential mechanisms.

This study has both strengths and limitations. By applying MR, it could hardly be affected by unobserved confounders and reverse causalities. We chose robust instrumental variables with F statistics that were larger than 10. We set the linkage disequilibrium clumping [LD] cutoff to r^2^ < 0.001 and < 1 MB from the index variant to guarantee the independence of the data. We set the genome-wide significance threshold to P = 5 × 10^−8^. Sensitivity analysis was also performed to ensure more reliable results, which involves examining the heterogeneity, pleiotropy and leave-one-SNP-out method. In addition, to make sure that no potential risk variables would contradict our findings, we also checked the Phenoscanncer (http://www.phenoscanner.medschl.cam.ac.uk/). However, the recruited individuals were all of European descent; therefore, it was unclear whether GERD and respiratory diseases were causally related in other groups. In addition, while our results simply described the causal relationship between GERD and respiratory diseases, the underlying mechanisms merit additional research.

## Conclusion

This is the first large-scale MR study to explore the relationship between GERD and respiratory diseases (COPD, bronchitis, pneumonia, lung cancer and pulmonary embolism). We found that GERD was causally related to a higher risk of respiratory diseases. Our research suggests physicians to give effective treatments for GERD on respiratory diseases. By exploring the gene expression, our study may also help to reveal the role played by the central nervous system and brain tissue in developing respiratory diseases caused by GERD.

### Supplementary Information

**Additional file 1: Table S1.** Genetic variants used in the analyses investigating a causal impact of genetically predicted GERD and respiratory diseases. **Table S2.** The allele frequencies in an average European population. **Figure S1.** MR Scatter plot for the genetic association between GERD and risk of COPD. **Figure S2.** MR Scatter plot for the genetic association between GERD and risk of bronchitis. **Figure S3.** MR Scatter plot for the genetic association between GERD and risk of pneumonia. **Figure S4.** MR Scatter plot for the genetic association between GERD and risk of lung cancer. **Figure S5.** MR Scatter plot for the genetic association between GERD and risk of pulmonary embolism. **Figure S6.** MR Funnel plot for the genetic association between GERD and risk of COPD. **Figure S7.** MR Funnel plot for the genetic association between GERD and risk of bronchitis. **Figure S8.** MR Funnel plot for the genetic association between GERD and risk of pneumonia. **Figure S9.** MR Funnel plot for the genetic association between GERD and risk of lung cancer. **Figure S10.** MR Funnel plot for the genetic association between GERD and risk of pulmonary embolism. **Figure S11.** MR Leave-one-SNP-out plot for the genetic association between GERD and risk of COPD. **Figure S12.** MR Leave-one-SNP-out plot for the genetic association between GERD and risk of bronchitis. **Figure S13.** MR Leave-one-SNP-out plot for the genetic association between GERD and risk of pneumonia. **Figure S14.** MR Leave-one-SNP-out plot for the genetic association between GERD and risk of lung cancer. **Figure S15.** MR Leave-one-SNP-out plot for the genetic association between GERD and risk of pulmonary embolism.

**Additional file 2. **Genes’ expression in normal tissues.

**Additional file 3: Table S3.** Genes’ expression in normal tissues.

## Data Availability

The data we used are publicly available summary statistics and can be obtained upon reasonable request.
