# Supplementary material for: Gastroesophageal reflux disease and the risk of respiratory diseases: a Mendelian randomization study
Source: J Transl Med. 2024 Jan 16;22:60. doi: 10.1186/s12967-023-04786-0 (PMC10790464; doi:10.1186/s12967-023-04786-0)

**Table S1** Genetic variants used in the analyses investigating a causal impact of genetically predicted GERD and respiratory diseases

| **SNP** | **EA** | **OA** | **EAF** | $\boldsymbol{\beta}$**_expsr_** | **SE_expsr_** | $\boldsymbol{P}$**_expsr_** | $\boldsymbol{\beta}$**_otcm_** | **SE_otcm_** | $\boldsymbol{P}$**_otcm_** | **R2** | **F** |
| --- | --- | --- | --- | --- | --- | --- | --- | --- | --- | --- | --- |
| **COPD on GERD** | | | | | | | | | | | |
| rs10010963 | T | C | 0.616433 | -0.02698 | 0.004947 | 4.92E-08 | -0.0266 | 0.0195 | 0.1738 | 0.000344 | 75.34104 |
| rs1011407 | G | A | 0.121628 | -0.04206 | 0.007359 | 1.09E-08 | -0.0159 | 0.0333 | 0.633501 | 0.000378 | 82.7396 |
| rs10133111 | A | G | 0.162996 | 0.041788 | 0.006508 | 1.35E-10 | -0.0201 | 0.0222 | 0.365 | 0.000476 | 104.2955 |
| rs1021363 | G | A | 0.641992 | -0.03122 | 0.005022 | 5.10E-10 | -0.0034 | 0.0207 | 0.8673 | 0.000448 | 98.05296 |
| rs10837002 | G | C | 0.35122 | 0.027649 | 0.005037 | 4.03E-08 | 0.033 | 0.0197 | 0.09307 | 0.000348 | 76.25204 |
| rs11762636 | A | C | 0.180282 | -0.05148 | 0.006256 | 1.88E-16 | 0.0045 | 0.0206 | 0.8291 | 0.000783 | 171.5305 |
| rs11953061 | T | C | 0.338908 | 0.02816 | 0.005087 | 3.10E-08 | 0.0256 | 0.0189 | 0.1756 | 0.000355 | 77.77172 |
| rs12204714 | T | C | 0.632223 | -0.02882 | 0.004994 | 7.92E-09 | 0.0153 | 0.0191 | 0.4229 | 0.000386 | 84.5243 |
| rs12357321 | A | G | 0.311087 | 0.031716 | 0.005231 | 1.33E-09 | 0.0568 | 0.0202 | 0.004843 | 0.000431 | 94.37328 |
| rs12453010 | T | C | 0.394803 | 0.029697 | 0.004933 | 1.75E-09 | 0.0486 | 0.019 | 0.0106 | 0.000421 | 92.24399 |
| rs12598916 | G | C | 0.274798 | -0.03326 | 0.005392 | 6.87E-10 | -0.0504 | 0.021 | 0.01663 | 0.000441 | 96.51762 |
| rs12967855 | G | A | 0.670435 | -0.03655 | 0.005134 | 1.09E-12 | -0.0118 | 0.0208 | 0.5697 | 0.00059 | 129.2034 |
| rs12997558 | A | G | 0.358751 | 0.027818 | 0.005022 | 3.04E-08 | -0.0276 | 0.0192 | 0.1498 | 0.000356 | 77.9292 |
| rs13107325 | T | C | 0.074445 | 0.070144 | 0.009183 | 2.20E-14 | 0.0116 | 0.0779 | 0.8816 | 0.000678 | 148.4494 |
| rs1334297 | A | G | 0.734249 | -0.0388 | 0.005455 | 1.14E-12 | -0.0231 | 0.0208 | 0.2653 | 0.000587 | 128.6064 |
| rs13409451 | G | A | 0.392403 | -0.02771 | 0.004932 | 1.93E-08 | -0.0189 | 0.0194 | 0.3313 | 0.000366 | 80.12755 |
| rs1431196 | G | A | 0.428432 | 0.03242 | 0.004864 | 2.65E-11 | -0.0064 | 0.0187 | 0.7327 | 0.000515 | 112.6816 |
| rs1479405 | T | C | 0.3217 | 0.031484 | 0.005151 | 9.85E-10 | 0.04 | 0.0201 | 0.04627 | 0.000433 | 94.69135 |
| rs1510719 | C | T | 0.383439 | -0.03888 | 0.004947 | 3.84E-15 | -0.0505 | 0.0191 | 0.008321 | 0.000715 | 156.5291 |
| rs1592757 | C | G | 0.355772 | 0.031105 | 0.005025 | 6.00E-10 | 0.0241 | 0.0193 | 0.2121 | 0.000444 | 97.07975 |
| rs1596747 | G | A | 0.494136 | 0.031087 | 0.004807 | 1.00E-10 | 0.0224 | 0.0184 | 0.2242 | 0.000483 | 105.7563 |
| rs1716171 | T | C | 0.790024 | 0.038398 | 0.005904 | 7.83E-11 | -0.0242 | 0.0225 | 0.2823 | 0.000489 | 107.0788 |
| rs17379561 | T | A | 0.144391 | 0.053071 | 0.006866 | 1.08E-14 | 0.0312 | 0.022 | 0.1554 | 0.000696 | 152.3703 |
| rs1883842 | G | T | 0.279255 | 0.030833 | 0.005368 | 9.27E-09 | 0.0125 | 0.0236 | 0.5975 | 0.000383 | 83.76208 |
| rs1937450 | G | T | 0.537739 | 0.031585 | 0.004845 | 7.07E-11 | 0.0301 | 0.0186 | 0.106 | 0.000496 | 108.5635 |
| rs2016933 | G | C | 0.730053 | -0.03103 | 0.005421 | 1.04E-08 | -0.0299 | 0.0216 | 0.1663 | 0.000379 | 83.04072 |
| rs2023878 | T | C | 0.192377 | -0.03628 | 0.006119 | 3.04E-09 | -0.036 | 0.021 | 0.08626 | 0.000409 | 89.54589 |
| rs2043539 | A | G | 0.41866 | 0.027206 | 0.004865 | 2.24E-08 | -0.0055 | 0.0191 | 0.7727 | 0.00036 | 78.85561 |
| rs215614 | A | G | 0.629725 | -0.03285 | 0.004977 | 4.08E-11 | -0.0346 | 0.0205 | 0.092321 | 0.000503 | 110.1881 |
| rs2164300 | T | C | 0.523279 | -0.02648 | 0.004827 | 4.13E-08 | -0.0212 | 0.0184 | 0.2489 | 0.00035 | 76.53959 |
| rs2240326 | A | G | 0.473775 | -0.04717 | 0.004813 | 1.13E-22 | -0.0086 | 0.0184 | 0.6428 | 0.001109 | 242.9875 |
| rs2396133 | G | A | 0.475329 | 0.029355 | 0.004818 | 1.11E-09 | 0.0133 | 0.0185 | 0.4713 | 0.00043 | 94.07729 |
| rs2396766 | A | G | 0.47308 | 0.032206 | 0.004819 | 2.34E-11 | 0.0134 | 0.0184 | 0.4666 | 0.000517 | 113.1959 |
| rs2734839 | T | C | 0.606693 | -0.02835 | 0.004928 | 8.79E-09 | -0.0143 | 0.0184 | 0.4374 | 0.000384 | 83.9397 |
| rs2744961 | T | C | 0.358437 | 0.029201 | 0.005015 | 5.81E-09 | 0.0228 | 0.0195 | 0.2424 | 0.000392 | 85.83619 |
| rs2782641 | A | G | 0.612669 | 0.027088 | 0.004946 | 4.33E-08 | 0.0124 | 0.0189 | 0.511601 | 0.000348 | 76.22214 |
| rs2815749 | G | A | 0.800974 | 0.038877 | 0.006022 | 1.07E-10 | -0.0049 | 0.0265 | 0.8542 | 0.000482 | 105.4819 |
| rs2834005 | C | T | 0.315 | 0.0297 | 0.005173 | 9.42E-09 | 0.0212 | 0.0206 | 0.3038 | 0.000381 | 83.31669 |
| rs2838771 | C | G | 0.646721 | -0.0281 | 0.005066 | 2.91E-08 | -0.0376 | 0.0203 | 0.06354 | 0.000361 | 78.96163 |
| rs324769 | T | C | 0.449179 | -0.02677 | 0.004833 | 3.05E-08 | -0.0313 | 0.0188 | 0.09689 | 0.000355 | 77.61379 |
| rs329122 | A | G | 0.419631 | -0.02895 | 0.004884 | 3.05E-09 | -0.0305 | 0.0186 | 0.101 | 0.000408 | 89.37061 |
| rs3766823 | A | G | 0.171467 | 0.03936 | 0.006385 | 7.09E-10 | -0.0159 | 0.0223 | 0.4745 | 0.00044 | 96.34984 |
| rs3793577 | G | A | 0.538279 | 0.027031 | 0.00485 | 2.49E-08 | 0.0259 | 0.0185 | 0.1606 | 0.000363 | 79.4927 |
| rs3828917 | T | G | 0.041826 | 0.067111 | 0.012005 | 2.27E-08 | 0.0098 | 0.075 | 0.8955 | 0.000361 | 79.01331 |
| rs3863241 | T | C | 0.52696 | 0.032498 | 0.004815 | 1.49E-11 | 0.0146 | 0.0187 | 0.4348 | 0.000527 | 115.2615 |
| rs4300861 | T | C | 0.38208 | 0.030713 | 0.004949 | 5.43E-10 | 0.0255 | 0.0186 | 0.1705 | 0.000445 | 97.49708 |
| rs4382592 | G | T | 0.699524 | -0.03027 | 0.005251 | 8.20E-09 | -0.0296 | 0.0206 | 0.15 | 0.000385 | 84.29576 |
| rs4713692 | T | C | 0.367808 | -0.02761 | 0.004986 | 3.07E-08 | 0.0077 | 0.0194 | 0.691301 | 0.000355 | 77.608 |
| rs569356 | G | A | 0.140835 | -0.03792 | 0.00691 | 4.07E-08 | -0.0674 | 0.0274 | 0.01409 | 0.000348 | 76.15764 |
| rs6711584 | A | G | 0.452019 | 0.032255 | 0.00484 | 2.66E-11 | 0.0234 | 0.0185 | 0.2056 | 0.000515 | 112.8205 |
| rs6722661 | A | G | 0.364669 | -0.03225 | 0.005004 | 1.15E-10 | -0.0238 | 0.0193 | 0.2182 | 0.000482 | 105.5225 |
| rs6780459 | T | A | 0.746622 | 0.030551 | 0.005521 | 3.14E-08 | 0.0065 | 0.0225 | 0.772999 | 0.000353 | 77.29063 |
| rs7032155 | A | C | 0.59185 | 0.02775 | 0.004914 | 1.63E-08 | 0.0227 | 0.019 | 0.2334 | 0.000372 | 81.42926 |
| rs7206608 | G | C | 0.322927 | 0.029154 | 0.005145 | 1.46E-08 | -0.0035 | 0.0192 | 0.8549 | 0.000372 | 81.35083 |
| rs7241572 | A | G | 0.209101 | 0.036551 | 0.005975 | 9.49E-10 | 0.0273 | 0.0224 | 0.2236 | 0.000442 | 96.72337 |
| rs7527682 | G | A | 0.53725 | -0.02668 | 0.004822 | 3.13E-08 | 6.00E-04 | 0.0189 | 0.9734 | 0.000354 | 77.48898 |
| rs7541875 | G | A | 0.426069 | 0.027397 | 0.00485 | 1.61E-08 | 0.0278 | 0.0188 | 0.1392 | 0.000367 | 80.34759 |
| rs7600261 | T | C | 0.306391 | 0.033803 | 0.005221 | 9.47E-11 | 8.00E-04 | 0.02 | 0.9671 | 0.000486 | 106.3124 |
| rs7612999 | A | G | 0.245338 | 0.030523 | 0.005595 | 4.90E-08 | 0.0225 | 0.0218 | 0.3022 | 0.000345 | 75.50677 |
| rs761777 | G | A | 0.254034 | 0.034534 | 0.005545 | 4.71E-10 | -0.0047 | 0.0207 | 0.8208 | 0.000452 | 98.93841 |
| rs7675588 | A | C | 0.794635 | -0.03352 | 0.005954 | 1.80E-08 | -0.0094 | 0.0247 | 0.7052 | 0.000367 | 80.2778 |
| rs7685686 | G | A | 0.422353 | -0.02792 | 0.004892 | 1.14E-08 | -0.0412 | 0.0185 | 0.02571 | 0.00038 | 83.2653 |
| rs773109 | A | G | 0.335269 | -0.03806 | 0.005102 | 8.71E-14 | -0.0102 | 0.02 | 0.611799 | 0.000646 | 141.3367 |
| rs7942368 | T | C | 0.214659 | -0.03397 | 0.005919 | 9.54E-09 | 0.012 | 0.0227 | 0.5956 | 0.000389 | 85.15015 |
| rs903678 | A | G | 0.339412 | 0.027738 | 0.005085 | 4.89E-08 | 0.0247 | 0.0202 | 0.2199 | 0.000345 | 75.51152 |
| rs903959 | A | T | 0.399262 | 0.029163 | 0.004916 | 2.99E-09 | -0.0159 | 0.0189 | 0.3997 | 0.000408 | 89.29952 |
| rs9372625 | A | G | 0.383042 | -0.03773 | 0.004954 | 2.62E-14 | -0.0195 | 0.0197 | 0.3233 | 0.000673 | 147.2856 |
| rs9373363 | G | A | 0.253631 | -0.03268 | 0.00556 | 4.13E-09 | -0.0606 | 0.0196 | 0.001993 | 0.000404 | 88.52233 |
| rs9396740 | A | G | 0.248794 | -0.03149 | 0.005559 | 1.47E-08 | -0.033 | 0.0229 | 0.1489 | 0.000371 | 81.14271 |
| rs942065 | A | G | 0.634045 | 0.030738 | 0.005009 | 8.45E-10 | 0.0017 | 0.019 | 0.9306 | 0.000438 | 95.9759 |
| rs9529055 | A | G | 0.475633 | 0.02666 | 0.004816 | 3.11E-08 | 0.028 | 0.0184 | 0.1293 | 0.000355 | 77.59899 |
| rs9542729 | G | C | 0.20244 | -0.03632 | 0.005999 | 1.41E-09 | -0.0342 | 0.0207 | 0.098431 | 0.000426 | 93.23597 |
| rs9615905 | T | C | 0.458193 | 0.027566 | 0.004838 | 1.21E-08 | 0.0034 | 0.0185 | 0.8551 | 0.000377 | 82.57649 |
| rs9636202 | A | G | 0.26663 | -0.03504 | 0.005472 | 1.51E-10 | -0.0554 | 0.0221 | 0.01229 | 0.00048 | 105.1307 |
| rs9940128 | A | G | 0.421755 | 0.033251 | 0.004863 | 8.06E-12 | 0.0108 | 0.0186 | 0.5616 | 0.000539 | 118.0552 |
| **Bronchitis on GERD** | | | | | | | | | |  |  |
| rs10010963 | T | C | 0.616433 | -0.02698 | 0.004947 | 4.92E-08 | -0.0092 | 0.0101 | 0.3629 | 0.000344 | 75.34104 |
| rs1011407 | G | A | 0.121628 | -0.04206 | 0.007359 | 1.09E-08 | -0.0051 | 0.0173 | 0.7687 | 0.000378 | 82.7396 |
| rs10133111 | A | G | 0.162996 | 0.041788 | 0.006508 | 1.35E-10 | 0.003 | 0.0115 | 0.7977 | 0.000476 | 104.2955 |
| rs1021363 | G | A | 0.641992 | -0.03122 | 0.005022 | 5.10E-10 | -0.0137 | 0.0107 | 0.2002 | 0.000448 | 98.05296 |
| rs10837002 | G | C | 0.35122 | 0.027649 | 0.005037 | 4.03E-08 | -0.0021 | 0.0102 | 0.8389 | 0.000348 | 76.25204 |
| rs11762636 | A | C | 0.180282 | -0.05148 | 0.006256 | 1.88E-16 | 0.0019 | 0.0107 | 0.8621 | 0.000783 | 171.5305 |
| rs11953061 | T | C | 0.338908 | 0.02816 | 0.005087 | 3.10E-08 | 0.0019 | 0.0099 | 0.8481 | 0.000355 | 77.77172 |
| rs12204714 | T | C | 0.632223 | -0.02882 | 0.004994 | 7.92E-09 | 0.0077 | 0.0099 | 0.435 | 0.000386 | 84.5243 |
| rs12357321 | A | G | 0.311087 | 0.031716 | 0.005231 | 1.33E-09 | -0.0053 | 0.0105 | 0.6132 | 0.000431 | 94.37328 |
| rs12453010 | T | C | 0.394803 | 0.029697 | 0.004933 | 1.75E-09 | 0.0132 | 0.0099 | 0.1812 | 0.000421 | 92.24399 |
| rs12598916 | G | C | 0.274798 | -0.03326 | 0.005392 | 6.87E-10 | -0.015 | 0.0109 | 0.1682 | 0.000441 | 96.51762 |
| rs12967855 | G | A | 0.670435 | -0.03655 | 0.005134 | 1.09E-12 | -0.0075 | 0.0108 | 0.4914 | 0.00059 | 129.2034 |
| rs12997558 | A | G | 0.358751 | 0.027818 | 0.005022 | 3.04E-08 | 0.003 | 0.01 | 0.7617 | 0.000356 | 77.9292 |
| rs13107325 | T | C | 0.074445 | 0.070144 | 0.009183 | 2.20E-14 | 0.01 | 0.0402 | 0.8036 | 0.000678 | 148.4494 |
| rs1334297 | A | G | 0.734249 | -0.0388 | 0.005455 | 1.14E-12 | 0.0011 | 0.0108 | 0.9215 | 0.000587 | 128.6064 |
| rs13409451 | G | A | 0.392403 | -0.02771 | 0.004932 | 1.93E-08 | -0.0164 | 0.0101 | 0.1039 | 0.000366 | 80.12755 |
| rs1431196 | G | A | 0.428432 | 0.03242 | 0.004864 | 2.65E-11 | 0.0209 | 0.0097 | 0.03119 | 0.000515 | 112.6816 |
| rs1479405 | T | C | 0.3217 | 0.031484 | 0.005151 | 9.85E-10 | 0.0016 | 0.0105 | 0.8766 | 0.000433 | 94.69135 |
| rs1510719 | C | T | 0.383439 | -0.03888 | 0.004947 | 3.84E-15 | -0.0021 | 0.0099 | 0.8303 | 0.000715 | 156.5291 |
| rs1592757 | C | G | 0.355772 | 0.031105 | 0.005025 | 6.00E-10 | 0.0117 | 0.01 | 0.2436 | 0.000444 | 97.07975 |
| rs1596747 | G | A | 0.494136 | 0.031087 | 0.004807 | 1.00E-10 | 0.0013 | 0.0096 | 0.8894 | 0.000483 | 105.7563 |
| rs1716171 | T | C | 0.790024 | 0.038398 | 0.005904 | 7.83E-11 | ######## | 0.0117 | 0.9505 | 0.000489 | 107.0788 |
| rs17379561 | T | A | 0.144391 | 0.053071 | 0.006866 | 1.08E-14 | 0.0046 | 0.0114 | 0.690601 | 0.000696 | 152.3703 |
| rs1883842 | G | T | 0.279255 | 0.030833 | 0.005368 | 9.27E-09 | 0.0167 | 0.0123 | 0.175 | 0.000383 | 83.76208 |
| rs1937450 | G | T | 0.537739 | 0.031585 | 0.004845 | 7.07E-11 | 0.0154 | 0.0097 | 0.1116 | 0.000496 | 108.5635 |
| rs2016933 | G | C | 0.730053 | -0.03103 | 0.005421 | 1.04E-08 | -0.0105 | 0.0112 | 0.3507 | 0.000379 | 83.04072 |
| rs2023878 | T | C | 0.192377 | -0.03628 | 0.006119 | 3.04E-09 | -0.0025 | 0.0109 | 0.818 | 0.000409 | 89.54589 |
| rs2043539 | A | G | 0.41866 | 0.027206 | 0.004865 | 2.24E-08 | 0.0046 | 0.0099 | 0.6446 | 0.00036 | 78.85561 |
| rs215614 | A | G | 0.629725 | -0.03285 | 0.004977 | 4.08E-11 | -0.0052 | 0.0107 | 0.6268 | 0.000503 | 110.1881 |
| rs2164300 | T | C | 0.523279 | -0.02648 | 0.004827 | 4.13E-08 | 5.00E-04 | 0.0096 | 0.961 | 0.00035 | 76.53959 |
| rs2240326 | A | G | 0.473775 | -0.04717 | 0.004813 | 1.13E-22 | -0.0183 | 0.0096 | 0.05532 | 0.001109 | 242.9875 |
| rs2396133 | G | A | 0.475329 | 0.029355 | 0.004818 | 1.11E-09 | -0.0032 | 0.0096 | 0.7396 | 0.00043 | 94.07729 |
| rs2396766 | A | G | 0.47308 | 0.032206 | 0.004819 | 2.34E-11 | -0.005 | 0.0096 | 0.6025 | 0.000517 | 113.1959 |
| rs2734839 | T | C | 0.606693 | -0.02835 | 0.004928 | 8.79E-09 | -0.0047 | 0.0096 | 0.6206 | 0.000384 | 83.9397 |
| rs2744961 | T | C | 0.358437 | 0.029201 | 0.005015 | 5.81E-09 | 0.0044 | 0.0101 | 0.6612 | 0.000392 | 85.83619 |
| rs2782641 | A | G | 0.612669 | 0.027088 | 0.004946 | 4.33E-08 | 0.0025 | 0.0098 | 0.7968 | 0.000348 | 76.22214 |
| rs2815749 | G | A | 0.800974 | 0.038877 | 0.006022 | 1.07E-10 | 0.0022 | 0.0138 | 0.8741 | 0.000482 | 105.4819 |
| rs2834005 | C | T | 0.315 | 0.0297 | 0.005173 | 9.42E-09 | 0.0225 | 0.0107 | 0.03559 | 0.000381 | 83.31669 |
| rs2838771 | C | G | 0.646721 | -0.0281 | 0.005066 | 2.91E-08 | -0.0074 | 0.0105 | 0.4821 | 0.000361 | 78.96163 |
| rs324769 | T | C | 0.449179 | -0.02677 | 0.004833 | 3.05E-08 | -0.0144 | 0.0098 | 0.1416 | 0.000355 | 77.61379 |
| rs329122 | A | G | 0.419631 | -0.02895 | 0.004884 | 3.05E-09 | -0.0252 | 0.0097 | 0.009226 | 0.000408 | 89.37061 |
| rs3766823 | A | G | 0.171467 | 0.03936 | 0.006385 | 7.09E-10 | 0.006 | 0.0116 | 0.6036 | 0.00044 | 96.34984 |
| rs3793577 | G | A | 0.538279 | 0.027031 | 0.00485 | 2.49E-08 | 0.0053 | 0.0096 | 0.5807 | 0.000363 | 79.4927 |
| rs3828917 | T | G | 0.041826 | 0.067111 | 0.012005 | 2.27E-08 | 0.0483 | 0.0383 | 0.2072 | 0.000361 | 79.01331 |
| rs3863241 | T | C | 0.52696 | 0.032498 | 0.004815 | 1.49E-11 | 0.0099 | 0.0097 | 0.309 | 0.000527 | 115.2615 |
| rs4300861 | T | C | 0.38208 | 0.030713 | 0.004949 | 5.43E-10 | -0.0062 | 0.0096 | 0.5213 | 0.000445 | 97.49708 |
| rs4382592 | G | T | 0.699524 | -0.03027 | 0.005251 | 8.20E-09 | 0.0018 | 0.0107 | 0.8632 | 0.000385 | 84.29576 |
| rs4713692 | T | C | 0.367808 | -0.02761 | 0.004986 | 3.07E-08 | -0.0032 | 0.0101 | 0.7514 | 0.000355 | 77.608 |
| rs569356 | G | A | 0.140835 | -0.03792 | 0.00691 | 4.07E-08 | -0.0093 | 0.0142 | 0.5125 | 0.000348 | 76.15764 |
| rs6711584 | A | G | 0.452019 | 0.032255 | 0.00484 | 2.66E-11 | 0.0153 | 0.0096 | 0.1116 | 0.000515 | 112.8205 |
| rs6722661 | A | G | 0.364669 | -0.03225 | 0.005004 | 1.15E-10 | ######## | 0.01 | 0.9406 | 0.000482 | 105.5225 |
| rs6780459 | T | A | 0.746622 | 0.030551 | 0.005521 | 3.14E-08 | 0.0206 | 0.0117 | 0.07858 | 0.000353 | 77.29063 |
| rs7032155 | A | C | 0.59185 | 0.02775 | 0.004914 | 1.63E-08 | -0.0033 | 0.0099 | 0.7363 | 0.000372 | 81.42926 |
| rs7206608 | G | C | 0.322927 | 0.029154 | 0.005145 | 1.46E-08 | 0.0076 | 0.01 | 0.4479 | 0.000372 | 81.35083 |
| rs7241572 | A | G | 0.209101 | 0.036551 | 0.005975 | 9.49E-10 | 0.0111 | 0.0117 | 0.3406 | 0.000442 | 96.72337 |
| rs7527682 | G | A | 0.53725 | -0.02668 | 0.004822 | 3.13E-08 | 0.0056 | 0.0099 | 0.5673 | 0.000354 | 77.48898 |
| rs7541875 | G | A | 0.426069 | 0.027397 | 0.00485 | 1.61E-08 | -0.0073 | 0.0098 | 0.456 | 0.000367 | 80.34759 |
| rs7600261 | T | C | 0.306391 | 0.033803 | 0.005221 | 9.47E-11 | -0.0112 | 0.0104 | 0.2809 | 0.000486 | 106.3124 |
| rs7612999 | A | G | 0.245338 | 0.030523 | 0.005595 | 4.90E-08 | 0.0125 | 0.0113 | 0.2697 | 0.000345 | 75.50677 |
| rs761777 | G | A | 0.254034 | 0.034534 | 0.005545 | 4.71E-10 | -0.0141 | 0.0107 | 0.1906 | 0.000452 | 98.93841 |
| rs7675588 | A | C | 0.794635 | -0.03352 | 0.005954 | 1.80E-08 | -0.0089 | 0.0128 | 0.4863 | 0.000367 | 80.2778 |
| rs7685686 | G | A | 0.422353 | -0.02792 | 0.004892 | 1.14E-08 | -0.0131 | 0.0096 | 0.171 | 0.00038 | 83.2653 |
| rs773109 | A | G | 0.335269 | -0.03806 | 0.005102 | 8.71E-14 | 0.0017 | 0.0104 | 0.8714 | 0.000646 | 141.3367 |
| rs7942368 | T | C | 0.214659 | -0.03397 | 0.005919 | 9.54E-09 | 0.0103 | 0.0118 | 0.3814 | 0.000389 | 85.15015 |
| rs903678 | A | G | 0.339412 | 0.027738 | 0.005085 | 4.89E-08 | 0.0036 | 0.0105 | 0.7326 | 0.000345 | 75.51152 |
| rs903959 | A | T | 0.399262 | 0.029163 | 0.004916 | 2.99E-09 | 0.0127 | 0.0098 | 0.195 | 0.000408 | 89.29952 |
| rs9372625 | A | G | 0.383042 | -0.03773 | 0.004954 | 2.62E-14 | -0.0043 | 0.0103 | 0.6772 | 0.000673 | 147.2856 |
| rs9373363 | G | A | 0.253631 | -0.03268 | 0.00556 | 4.13E-09 | -0.0184 | 0.0102 | 0.069539 | 0.000404 | 88.52233 |
| rs9396740 | A | G | 0.248794 | -0.03149 | 0.005559 | 1.47E-08 | -0.0024 | 0.0119 | 0.8376 | 0.000371 | 81.14271 |
| rs942065 | A | G | 0.634045 | 0.030738 | 0.005009 | 8.45E-10 | 0.0189 | 0.0099 | 0.05663 | 0.000438 | 95.9759 |
| rs9529055 | A | G | 0.475633 | 0.02666 | 0.004816 | 3.11E-08 | 0.0124 | 0.0096 | 0.1939 | 0.000355 | 77.59899 |
| rs9542729 | G | C | 0.20244 | -0.03632 | 0.005999 | 1.41E-09 | 0.0083 | 0.0107 | 0.4375 | 0.000426 | 93.23597 |
| rs9615905 | T | C | 0.458193 | 0.027566 | 0.004838 | 1.21E-08 | 0.0197 | 0.0096 | 0.04005 | 0.000377 | 82.57649 |
| rs9636202 | A | G | 0.26663 | -0.03504 | 0.005472 | 1.51E-10 | 0.0041 | 0.0115 | 0.7229 | 0.00048 | 105.1307 |
| rs9940128 | A | G | 0.421755 | 0.033251 | 0.004863 | 8.06E-12 | 0.0118 | 0.0096 | 0.2208 | 0.000539 | 118.0552 |
| **Pneumonia on GERD** | | | | | | | | | |  |  |
| rs10010963 | T | C | 0.616433 | -0.02698 | 0.004947 | 4.92E-08 | -0.0062 | 0.0109 | 0.568499 | 0.000172 | 36.53924 |
| rs1011407 | G | A | 0.121628 | -0.04206 | 0.007359 | 1.09E-08 | -0.0452 | 0.0186 | 0.01484 | 0.000189 | 40.12674 |
| rs10133111 | A | G | 0.162996 | 0.041788 | 0.006508 | 1.35E-10 | 0.0175 | 0.0124 | 0.1576 | 0.000238 | 50.57837 |
| rs1021363 | G | A | 0.641992 | -0.03122 | 0.005022 | 5.10E-10 | -0.0116 | 0.0115 | 0.3105 | 0.000224 | 47.5517 |
| rs10837002 | G | C | 0.35122 | 0.027649 | 0.005037 | 4.03E-08 | -0.0082 | 0.011 | 0.454099 | 0.000174 | 36.98098 |
| rs11762636 | A | C | 0.180282 | -0.05148 | 0.006256 | 1.88E-16 | -0.008 | 0.0115 | 0.484299 | 0.000392 | 83.17134 |
| rs11953061 | T | C | 0.338908 | 0.02816 | 0.005087 | 3.10E-08 | 0.0101 | 0.0105 | 0.34 | 0.000178 | 37.71787 |
| rs12204714 | T | C | 0.632223 | -0.02882 | 0.004994 | 7.92E-09 | 0.0065 | 0.0106 | 0.5426 | 0.000193 | 40.99211 |
| rs12357321 | A | G | 0.311087 | 0.031716 | 0.005231 | 1.33E-09 | 0.0233 | 0.0112 | 0.03811 | 0.000216 | 45.76759 |
| rs12453010 | T | C | 0.394803 | 0.029697 | 0.004933 | 1.75E-09 | 0.0135 | 0.0106 | 0.201 | 0.000211 | 44.73518 |
| rs12598916 | G | C | 0.274798 | -0.03326 | 0.005392 | 6.87E-10 | -0.0132 | 0.0117 | 0.2593 | 0.00022 | 46.80728 |
| rs12967855 | G | A | 0.670435 | -0.03655 | 0.005134 | 1.09E-12 | 0.007 | 0.0116 | 0.5483 | 0.000295 | 62.65393 |
| rs12997558 | A | G | 0.358751 | 0.027818 | 0.005022 | 3.04E-08 | -0.0048 | 0.0107 | 0.6532 | 0.000178 | 37.79423 |
| rs13107325 | T | C | 0.074445 | 0.070144 | 0.009183 | 2.20E-14 | -0.0251 | 0.0434 | 0.562999 | 0.000339 | 71.98365 |
| rs1334297 | A | G | 0.734249 | -0.0388 | 0.005455 | 1.14E-12 | -0.0078 | 0.0115 | 0.4984 | 0.000294 | 62.36452 |
| rs13409451 | G | A | 0.392403 | -0.02771 | 0.004932 | 1.93E-08 | -0.0133 | 0.0108 | 0.2195 | 0.000183 | 38.8602 |
| rs1431196 | G | A | 0.428432 | 0.03242 | 0.004864 | 2.65E-11 | 0.0284 | 0.0104 | 0.006301 | 0.000257 | 54.64415 |
| rs1479405 | T | C | 0.3217 | 0.031484 | 0.005151 | 9.85E-10 | 0.0017 | 0.0112 | 0.8801 | 0.000216 | 45.92181 |
| rs1510719 | C | T | 0.383439 | -0.03888 | 0.004947 | 3.84E-15 | -0.011 | 0.0106 | 0.3001 | 0.000357 | 75.90012 |
| rs1592757 | C | G | 0.355772 | 0.031105 | 0.005025 | 6.00E-10 | -0.0053 | 0.0107 | 0.625101 | 0.000222 | 47.07984 |
| rs1596747 | G | A | 0.494136 | 0.031087 | 0.004807 | 1.00E-10 | ######## | 0.0102 | 0.9429 | 0.000242 | 51.28662 |
| rs1716171 | T | C | 0.790024 | 0.038398 | 0.005904 | 7.83E-11 | 6.00E-04 | 0.0125 | 0.9645 | 0.000245 | 51.92778 |
| rs17379561 | T | A | 0.144391 | 0.053071 | 0.006866 | 1.08E-14 | 0.0154 | 0.0122 | 0.2096 | 0.000348 | 73.88423 |
| rs1883842 | G | T | 0.279255 | 0.030833 | 0.005368 | 9.27E-09 | 0.0161 | 0.0131 | 0.2196 | 0.000191 | 40.62253 |
| rs1937450 | G | T | 0.537739 | 0.031585 | 0.004845 | 7.07E-11 | 0.0041 | 0.0103 | 0.6885 | 0.000248 | 52.6476 |
| rs2016933 | G | C | 0.730053 | -0.03103 | 0.005421 | 1.04E-08 | -0.0301 | 0.012 | 0.0123 | 0.00019 | 40.27275 |
| rs2023878 | T | C | 0.192377 | -0.03628 | 0.006119 | 3.04E-09 | 0.0029 | 0.0117 | 0.8011 | 0.000205 | 43.42696 |
| rs2043539 | A | G | 0.41866 | 0.027206 | 0.004865 | 2.24E-08 | 0.0113 | 0.0106 | 0.2875 | 0.00018 | 38.24344 |
| rs215614 | A | G | 0.629725 | -0.03285 | 0.004977 | 4.08E-11 | ######## | 0.0114 | 0.9393 | 0.000252 | 53.43525 |
| rs2164300 | T | C | 0.523279 | -0.02648 | 0.004827 | 4.13E-08 | -0.0022 | 0.0102 | 0.8282 | 0.000175 | 37.12041 |
| rs2240326 | A | G | 0.473775 | -0.04717 | 0.004813 | 1.13E-22 | -0.0056 | 0.0103 | 0.5856 | 0.000555 | 117.8 |
| rs2396133 | G | A | 0.475329 | 0.029355 | 0.004818 | 1.11E-09 | -0.0121 | 0.0103 | 0.2412 | 0.000215 | 45.62407 |
| rs2396766 | A | G | 0.47308 | 0.032206 | 0.004819 | 2.34E-11 | 0.0034 | 0.0102 | 0.743 | 0.000259 | 54.89353 |
| rs2734839 | T | C | 0.606693 | -0.02835 | 0.004928 | 8.79E-09 | -0.004 | 0.0103 | 0.7 | 0.000192 | 40.70865 |
| rs2744961 | T | C | 0.358437 | 0.029201 | 0.005015 | 5.81E-09 | 0.0115 | 0.0109 | 0.2887 | 0.000196 | 41.62823 |
| rs2782641 | A | G | 0.612669 | 0.027088 | 0.004946 | 4.33E-08 | 0.0081 | 0.0105 | 0.4428 | 0.000174 | 36.96648 |
| rs2815749 | G | A | 0.800974 | 0.038877 | 0.006022 | 1.07E-10 | -0.0187 | 0.0147 | 0.2052 | 0.000241 | 51.15355 |
| rs2834005 | C | T | 0.315 | 0.0297 | 0.005173 | 9.42E-09 | 0.0114 | 0.0115 | 0.322 | 0.00019 | 40.40657 |
| rs2838771 | C | G | 0.646721 | -0.0281 | 0.005066 | 2.91E-08 | -0.0044 | 0.0113 | 0.697999 | 0.00018 | 38.29485 |
| rs324769 | T | C | 0.449179 | -0.02677 | 0.004833 | 3.05E-08 | -0.0183 | 0.0105 | 0.08012 | 0.000177 | 37.64129 |
| rs329122 | A | G | 0.419631 | -0.02895 | 0.004884 | 3.05E-09 | 0.0022 | 0.0104 | 0.8341 | 0.000204 | 43.34197 |
| rs3766823 | A | G | 0.171467 | 0.03936 | 0.006385 | 7.09E-10 | 0.0095 | 0.0124 | 0.4439 | 0.00022 | 46.72593 |
| rs3793577 | G | A | 0.538279 | 0.027031 | 0.00485 | 2.49E-08 | -0.008 | 0.0103 | 0.4365 | 0.000182 | 38.55236 |
| rs3828917 | T | G | 0.041826 | 0.067111 | 0.012005 | 2.27E-08 | -0.0255 | 0.0413 | 0.536701 | 0.000181 | 38.31991 |
| rs3863241 | T | C | 0.52696 | 0.032498 | 0.004815 | 1.49E-11 | ######## | 0.0104 | 0.946 | 0.000263 | 55.89495 |
| rs4300861 | T | C | 0.38208 | 0.030713 | 0.004949 | 5.43E-10 | -0.0055 | 0.0103 | 0.5942 | 0.000223 | 47.28218 |
| rs4382592 | G | T | 0.699524 | -0.03027 | 0.005251 | 8.20E-09 | 0.0073 | 0.0114 | 0.5221 | 0.000193 | 40.8813 |
| rs4713692 | T | C | 0.367808 | -0.02761 | 0.004986 | 3.07E-08 | 0.0068 | 0.0108 | 0.5302 | 0.000177 | 37.63848 |
| rs569356 | G | A | 0.140835 | -0.03792 | 0.00691 | 4.07E-08 | -0.004 | 0.0152 | 0.7944 | 0.000174 | 36.93521 |
| rs6711584 | A | G | 0.452019 | 0.032255 | 0.00484 | 2.66E-11 | 0.0094 | 0.0103 | 0.3578 | 0.000258 | 54.71152 |
| rs6722661 | A | G | 0.364669 | -0.03225 | 0.005004 | 1.15E-10 | 0.0129 | 0.0107 | 0.2289 | 0.000241 | 51.17324 |
| rs6780459 | T | A | 0.746622 | 0.030551 | 0.005521 | 3.14E-08 | -0.002 | 0.0125 | 0.8722 | 0.000177 | 37.48459 |
| rs7032155 | A | C | 0.59185 | 0.02775 | 0.004914 | 1.63E-08 | 0.005 | 0.0106 | 0.6373 | 0.000186 | 39.49138 |
| rs7206608 | G | C | 0.322927 | 0.029154 | 0.005145 | 1.46E-08 | 0.0118 | 0.0107 | 0.2678 | 0.000186 | 39.45335 |
| rs7241572 | A | G | 0.209101 | 0.036551 | 0.005975 | 9.49E-10 | -0.01 | 0.0125 | 0.4215 | 0.000221 | 46.90704 |
| rs7527682 | G | A | 0.53725 | -0.02668 | 0.004822 | 3.13E-08 | -0.0036 | 0.0105 | 0.7312 | 0.000177 | 37.58077 |
| rs7541875 | G | A | 0.426069 | 0.027397 | 0.00485 | 1.61E-08 | -0.0068 | 0.0105 | 0.5142 | 0.000184 | 38.96689 |
| rs7600261 | T | C | 0.306391 | 0.033803 | 0.005221 | 9.47E-11 | -0.0058 | 0.0111 | 0.6025 | 0.000243 | 51.55622 |
| rs7612999 | A | G | 0.245338 | 0.030523 | 0.005595 | 4.90E-08 | 0.0154 | 0.0121 | 0.2051 | 0.000172 | 36.6196 |
| rs761777 | G | A | 0.254034 | 0.034534 | 0.005545 | 4.71E-10 | 0.0114 | 0.0115 | 0.3208 | 0.000226 | 47.98101 |
| rs7675588 | A | C | 0.794635 | -0.03352 | 0.005954 | 1.80E-08 | -0.0086 | 0.0138 | 0.5337 | 0.000183 | 38.93305 |
| rs7685686 | G | A | 0.422353 | -0.02792 | 0.004892 | 1.14E-08 | -0.005 | 0.0103 | 0.6297 | 0.00019 | 40.38165 |
| rs773109 | A | G | 0.335269 | -0.03806 | 0.005102 | 8.71E-14 | 0.0057 | 0.0111 | 0.611899 | 0.000323 | 68.53577 |
| rs7942368 | T | C | 0.214659 | -0.03397 | 0.005919 | 9.54E-09 | -0.005 | 0.0126 | 0.690799 | 0.000195 | 41.29558 |
| rs903678 | A | G | 0.339412 | 0.027738 | 0.005085 | 4.89E-08 | 0.009 | 0.0112 | 0.425 | 0.000173 | 36.6219 |
| rs903959 | A | T | 0.399262 | 0.029163 | 0.004916 | 2.99E-09 | 0.009 | 0.0105 | 0.3913 | 0.000204 | 43.3075 |
| rs9372625 | A | G | 0.383042 | -0.03773 | 0.004954 | 2.62E-14 | -0.0248 | 0.011 | 0.02395 | 0.000336 | 71.41948 |
| rs9373363 | G | A | 0.253631 | -0.03268 | 0.00556 | 4.13E-09 | 0.0049 | 0.0109 | 0.6519 | 0.000202 | 42.93066 |
| rs9396740 | A | G | 0.248794 | -0.03149 | 0.005559 | 1.47E-08 | -0.0109 | 0.0127 | 0.3921 | 0.000185 | 39.35243 |
| rs942065 | A | G | 0.634045 | 0.030738 | 0.005009 | 8.45E-10 | 0 | 0.0106 | 1 | 0.000219 | 46.54463 |
| rs9529055 | A | G | 0.475633 | 0.02666 | 0.004816 | 3.11E-08 | 0.0027 | 0.0103 | 0.7939 | 0.000177 | 37.63412 |
| rs9542729 | G | C | 0.20244 | -0.03632 | 0.005999 | 1.41E-09 | -0.0176 | 0.0115 | 0.125 | 0.000213 | 45.21615 |
| rs9615905 | T | C | 0.458193 | 0.027566 | 0.004838 | 1.21E-08 | ######## | 0.0103 | 0.9956 | 0.000189 | 40.04766 |
| rs9636202 | A | G | 0.26663 | -0.03504 | 0.005472 | 1.51E-10 | -0.0019 | 0.0123 | 0.8753 | 0.00024 | 50.98329 |
| rs9940128 | A | G | 0.421755 | 0.033251 | 0.004863 | 8.06E-12 | 0.0068 | 0.0103 | 0.5125 | 0.00027 | 57.24935 |
| **Lung cancer on GERD** | | | | | | | | | |  |  |
| rs1011407 | G | A | 0.121628 | -0.042062 | 0.0073592 | 1.09E-08 | 5.85E-05 | 0.121004 | 0.0002982 | 0.000378 | 141.69371 |
| rs10133111 | A | G | 0.162996 | 0.0417875 | 0.0065079 | 1.35E-10 | 1.30E-05 | 0.163557 | 0.0002624 | 0.0004765 | 178.6088 |
| rs1021363 | G | A | 0.641992 | -0.031217 | 0.005022 | 5.10E-10 | 0.0001018 | 0.643521 | 0.0002032 | 0.000448 | 167.91824 |
| rs10837002 | G | C | 0.35122 | 0.0276491 | 0.0050367 | 4.03E-08 | -6.64E-05 | 0.352409 | 0.0002032 | 0.0003484 | 130.5836 |
| rs11762636 | A | C | 0.180282 | -0.051483 | 0.006256 | 1.88E-16 | -0.000379 | 0.18239 | 0.0002519 | 0.0007834 | 293.75038 |
| rs11953061 | T | C | 0.338908 | 0.0281599 | 0.0050869 | 3.10E-08 | -0.000182 | 0.338012 | 0.0002063 | 0.0003553 | 133.1861 |
| rs12204714 | T | C | 0.632223 | -0.028817 | 0.0049942 | 7.92E-09 | 8.05E-05 | 0.6325 | 0.0002017 | 0.0003862 | 144.75006 |
| rs12357321 | A | G | 0.311087 | 0.0317159 | 0.0052308 | 1.33E-09 | 4.14E-05 | 0.312342 | 0.0002115 | 0.0004312 | 161.6167 |
| rs12453010 | T | C | 0.394803 | 0.0296967 | 0.0049332 | 1.75E-09 | 0.0002347 | 0.393375 | 0.0001997 | 0.0004214 | 157.97024 |
| rs12598916 | G | C | 0.274798 | -0.033261 | 0.0053917 | 6.87E-10 | 0.0001683 | 0.275271 | 0.000218 | 0.0004409 | 165.28893 |
| rs12997558 | A | G | 0.358751 | 0.0278184 | 0.0050221 | 3.04E-08 | 9.98E-05 | 0.358982 | 0.0002032 | 0.0003561 | 133.45578 |
| rs1334297 | A | G | 0.734249 | -0.038798 | 0.0054551 | 1.14E-12 | -0.000359 | 0.735623 | 0.0002208 | 0.0005875 | 220.24179 |
| rs13409451 | G | A | 0.392403 | -0.027708 | 0.0049318 | 1.93E-08 | 6.89E-05 | 0.390247 | 0.0001996 | 0.0003661 | 137.22052 |
| rs1431196 | G | A | 0.428432 | 0.0324197 | 0.0048645 | 2.65E-11 | -0.000246 | 0.426244 | 0.0001969 | 0.0005148 | 192.9701 |
| rs1479405 | T | C | 0.3217 | 0.0314843 | 0.0051514 | 9.85E-10 | -0.000284 | 0.322107 | 0.0002081 | 0.0004326 | 162.16141 |
| rs1592757 | C | G | 0.355772 | 0.0311051 | 0.0050247 | 6.00E-10 | 6.50E-05 | 0.356111 | 0.000203 | 0.0004435 | 166.2516 |
| rs1596747 | G | A | 0.494136 | 0.0310869 | 0.0048074 | 1.00E-10 | 0.0001741 | 0.492385 | 0.0001946 | 0.0004831 | 181.11049 |
| rs1716171 | T | C | 0.790024 | 0.0383981 | 0.0059038 | 7.83E-11 | 0.0001125 | 0.787905 | 0.0002382 | 0.0004892 | 183.37517 |
| rs17379561 | T | A | 0.144391 | 0.0530714 | 0.0068657 | 1.08E-14 | 0.0003516 | 0.142589 | 0.000278 | 0.0006959 | 260.93812 |
| rs1883842 | G | T | 0.279255 | 0.0308332 | 0.0053683 | 9.27E-09 | 0.0003999 | 0.277 | 0.0002175 | 0.0003827 | 143.44475 |
| rs1937450 | G | T | 0.537739 | 0.0315845 | 0.0048448 | 7.07E-11 | 0.0001761 | 0.534791 | 0.0001966 | 0.0004959 | 185.91775 |
| rs2016933 | G | C | 0.730053 | -0.031025 | 0.0054206 | 1.04E-08 | -0.000223 | 0.726316 | 0.0002181 | 0.0003794 | 142.2094 |
| rs2023878 | T | C | 0.192377 | -0.036285 | 0.0061191 | 3.04E-09 | 2.60E-05 | 0.192222 | 0.0002473 | 0.0004091 | 153.34967 |
| rs2043539 | A | G | 0.41866 | 0.0272058 | 0.0048649 | 2.24E-08 | 1.17E-05 | 0.419412 | 0.0001967 | 0.0003603 | 135.04229 |
| rs2106353 | T | G | 0.231451 | 0.0367491 | 0.0057251 | 1.37E-10 | 0.0002032 | 0.230287 | 0.0002318 | 0.0004805 | 180.10732 |
| rs2164300 | T | C | 0.523279 | -0.026475 | 0.0048268 | 4.13E-08 | -7.68E-06 | 0.518823 | 0.0001952 | 0.0003497 | 131.07605 |
| rs2240326 | A | G | 0.473775 | -0.047168 | 0.0048134 | 1.13E-22 | -0.000241 | 0.476921 | 0.0001945 | 0.0011094 | 416.12236 |
| rs2396133 | G | A | 0.475329 | 0.0293547 | 0.0048179 | 1.11E-09 | -0.000299 | 0.476802 | 0.000195 | 0.0004298 | 161.10981 |
| rs2396766 | A | G | 0.47308 | 0.0322057 | 0.0048188 | 2.34E-11 | 0.0001428 | 0.469737 | 0.0001947 | 0.0005171 | 193.85098 |
| rs2734839 | T | C | 0.606693 | -0.028348 | 0.0049278 | 8.79E-09 | -0.000485 | 0.607025 | 0.0001993 | 0.0003835 | 143.74892 |
| rs2834005 | C | T | 0.315 | 0.0296997 | 0.0051734 | 9.42E-09 | -0.000155 | 0.314881 | 0.0002091 | 0.0003807 | 142.68201 |
| rs2838771 | C | G | 0.646721 | -0.028098 | 0.0050657 | 2.91E-08 | -0.000277 | 0.646152 | 0.0002037 | 0.0003608 | 135.22385 |
| rs324769 | T | C | 0.449179 | -0.02677 | 0.0048333 | 3.05E-08 | 0.0001725 | 0.445618 | 0.0001956 | 0.0003546 | 132.91564 |
| rs3766823 | A | G | 0.171467 | 0.0393599 | 0.0063855 | 7.09E-10 | 0.0005978 | 0.171818 | 0.0002577 | 0.0004402 | 165.0016 |
| rs3793577 | G | A | 0.538279 | 0.0270309 | 0.0048498 | 2.49E-08 | 4.41E-05 | 0.53675 | 0.0001969 | 0.0003632 | 136.13333 |
| rs3828917 | T | G | 0.041826 | 0.0671113 | 0.0120054 | 2.27E-08 | 0.0009005 | 0.041611 | 0.0004861 | 0.000361 | 135.31235 |
| rs3863241 | T | C | 0.52696 | 0.0324982 | 0.0048152 | 1.49E-11 | 0.0002762 | 0.528113 | 0.0001947 | 0.0005265 | 197.38833 |
| rs4300861 | T | C | 0.38208 | 0.0307132 | 0.0049489 | 5.43E-10 | 0.0001496 | 0.382134 | 0.0002005 | 0.0004454 | 166.96629 |
| rs4382592 | G | T | 0.699524 | -0.030268 | 0.0052509 | 8.20E-09 | -0.000433 | 0.69876 | 0.0002122 | 0.0003851 | 144.35868 |
| rs4713692 | T | C | 0.367808 | -0.027613 | 0.0049863 | 3.07E-08 | -0.000168 | 0.36452 | 0.0002017 | 0.0003546 | 132.90573 |
| rs569356 | G | A | 0.140835 | -0.037919 | 0.0069097 | 4.07E-08 | 0.0004449 | 0.140053 | 0.00028 | 0.000348 | 130.42195 |
| rs6722661 | A | G | 0.364669 | -0.032254 | 0.0050035 | 1.15E-10 | -0.0001 | 0.362052 | 0.0002025 | 0.0004821 | 180.70999 |
| rs6780459 | T | A | 0.746622 | 0.0305507 | 0.0055211 | 3.14E-08 | 6.69E-05 | 0.747165 | 0.0002239 | 0.0003531 | 132.36222 |
| rs7032155 | A | C | 0.59185 | 0.02775 | 0.0049136 | 1.63E-08 | 0.0001066 | 0.591671 | 0.0001989 | 0.000372 | 139.44972 |
| rs7206608 | G | C | 0.322927 | 0.0291541 | 0.005145 | 1.46E-08 | 0.0004194 | 0.32149 | 0.0002083 | 0.0003717 | 139.31542 |
| rs7241572 | A | G | 0.209101 | 0.0365511 | 0.0059746 | 9.49E-10 | 0.0002068 | 0.207948 | 0.0002429 | 0.0004419 | 165.64129 |
| rs7527682 | G | A | 0.53725 | -0.026684 | 0.0048217 | 3.13E-08 | -0.000199 | 0.537131 | 0.0001951 | 0.000354 | 132.70189 |
| rs7541875 | G | A | 0.426069 | 0.0273972 | 0.0048498 | 1.61E-08 | 0.0001976 | 0.425246 | 0.0001963 | 0.0003671 | 137.59735 |
| rs7600261 | T | C | 0.306391 | 0.0338034 | 0.0052205 | 9.47E-11 | 0.0002953 | 0.306342 | 0.0002113 | 0.0004857 | 182.06275 |
| rs7612999 | A | G | 0.245338 | 0.0305231 | 0.0055955 | 4.90E-08 | 6.08E-05 | 0.244378 | 0.0002264 | 0.000345 | 129.3073 |
| rs761777 | G | A | 0.254034 | 0.0345341 | 0.0055447 | 4.71E-10 | -5.68E-05 | 0.254707 | 0.0002228 | 0.000452 | 169.43461 |
| rs7675588 | A | C | 0.794635 | -0.033523 | 0.0059544 | 1.80E-08 | -0.000218 | 0.793381 | 0.0002403 | 0.0003668 | 137.47782 |
| rs7685686 | G | A | 0.422353 | -0.027922 | 0.0048919 | 1.14E-08 | -0.000257 | 0.422552 | 0.0001969 | 0.0003804 | 142.594 |
| rs773109 | A | G | 0.335269 | -0.038057 | 0.0051021 | 8.71E-14 | -0.000403 | 0.33484 | 0.000206 | 0.0006456 | 242.04275 |
| rs7942368 | T | C | 0.214659 | -0.033968 | 0.0059192 | 9.54E-09 | 0.0004584 | 0.217696 | 0.0002386 | 0.000389 | 145.82185 |
| rs903959 | A | T | 0.399262 | 0.0291631 | 0.0049161 | 2.99E-09 | 4.20E-05 | 0.397636 | 0.000199 | 0.000408 | 152.92775 |
| rs9372625 | A | G | 0.383042 | -0.037727 | 0.0049537 | 2.62E-14 | -4.25E-05 | 0.381634 | 0.0002007 | 0.0006727 | 252.23039 |
| rs9373363 | G | A | 0.253631 | -0.032684 | 0.0055597 | 4.13E-09 | 0.0002893 | 0.252202 | 0.0002255 | 0.0004044 | 151.59679 |
| rs9396740 | A | G | 0.248794 | -0.031493 | 0.0055587 | 1.47E-08 | -0.000147 | 0.251188 | 0.0002242 | 0.0003707 | 138.959 |
| rs942065 | A | G | 0.634045 | 0.0307384 | 0.0050093 | 8.45E-10 | -8.63E-05 | 0.633171 | 0.0002024 | 0.0004385 | 164.36123 |
| rs9517313 | C | G | 0.383217 | 0.0331144 | 0.0049405 | 2.05E-11 | 0.0001731 | 0.380002 | 0.0002002 | 0.0005184 | 194.32772 |
| rs9529055 | A | G | 0.475633 | 0.0266604 | 0.0048164 | 3.11E-08 | 0.000107 | 0.476282 | 0.0001946 | 0.0003545 | 132.8903 |
| rs9542729 | G | C | 0.20244 | -0.036319 | 0.0059991 | 1.41E-09 | 0.0002526 | 0.202156 | 0.0002426 | 0.000426 | 159.66903 |
| rs9615905 | T | C | 0.458193 | 0.0275657 | 0.0048378 | 1.21E-08 | 1.62E-05 | 0.456685 | 0.0001951 | 0.0003773 | 141.41439 |
| rs9636202 | A | G | 0.26663 | -0.035044 | 0.0054719 | 1.51E-10 | 0.0004912 | 0.267353 | 0.0002204 | 0.0004803 | 180.03906 |
| **Pulmonary embolism on GERD** | | | | | | | | | |  |  |
| rs10010963 | T | C | 0.616433 | -0.02698 | 0.004947 | 4.92E-08 | -0.0064 | 0.0241 | 0.7921 | 0.000344 | 75.21053 |
| rs1011407 | G | A | 0.121628 | -0.04206 | 0.007359 | 1.09E-08 | 0.0207 | 0.0412 | 0.616 | 0.000378 | 82.59627 |
| rs10133111 | A | G | 0.162996 | 0.041788 | 0.006508 | 1.35E-10 | -0.0044 | 0.0275 | 0.8714 | 0.000476 | 104.1149 |
| rs1021363 | G | A | 0.641992 | -0.03122 | 0.005022 | 5.10E-10 | -0.0145 | 0.0255 | 0.568 | 0.000448 | 97.88311 |
| rs10837002 | G | C | 0.35122 | 0.027649 | 0.005037 | 4.03E-08 | -0.0198 | 0.0243 | 0.4159 | 0.000348 | 76.11995 |
| rs11762636 | A | C | 0.180282 | -0.05148 | 0.006256 | 1.88E-16 | -0.024 | 0.0255 | 0.3461 | 0.000783 | 171.2333 |
| rs11953061 | T | C | 0.338908 | 0.02816 | 0.005087 | 3.10E-08 | 0.0139 | 0.0234 | 0.5538 | 0.000355 | 77.637 |
| rs12204714 | T | C | 0.632223 | -0.02882 | 0.004994 | 7.92E-09 | 0.0292 | 0.0236 | 0.2166 | 0.000386 | 84.37788 |
| rs12357321 | A | G | 0.311087 | 0.031716 | 0.005231 | 1.33E-09 | 0.023 | 0.025 | 0.3558 | 0.000431 | 94.2098 |
| rs12453010 | T | C | 0.394803 | 0.029697 | 0.004933 | 1.75E-09 | -0.0294 | 0.0235 | 0.2114 | 0.000421 | 92.0842 |
| rs12598916 | G | C | 0.274798 | -0.03326 | 0.005392 | 6.87E-10 | 0.0348 | 0.0259 | 0.1792 | 0.000441 | 96.35042 |
| rs12967855 | G | A | 0.670435 | -0.03655 | 0.005134 | 1.09E-12 | 0.0023 | 0.0257 | 0.9289 | 0.00059 | 128.9796 |
| rs12997558 | A | G | 0.358751 | 0.027818 | 0.005022 | 3.04E-08 | -0.017 | 0.0237 | 0.473301 | 0.000356 | 77.79421 |
| rs13107325 | T | C | 0.074445 | 0.070144 | 0.009183 | 2.20E-14 | 0.0558 | 0.0954 | 0.5586 | 0.000678 | 148.1923 |
| rs1334297 | A | G | 0.734249 | -0.0388 | 0.005455 | 1.14E-12 | 0.0206 | 0.0257 | 0.4235 | 0.000587 | 128.3836 |
| rs13409451 | G | A | 0.392403 | -0.02771 | 0.004932 | 1.93E-08 | -0.0148 | 0.024 | 0.5364 | 0.000366 | 79.98875 |
| rs1431196 | G | A | 0.428432 | 0.03242 | 0.004864 | 2.65E-11 | 0.0353 | 0.0231 | 0.126 | 0.000515 | 112.4864 |
| rs1479405 | T | C | 0.3217 | 0.031484 | 0.005151 | 9.85E-10 | 0.0585 | 0.0248 | 0.01828 | 0.000433 | 94.52732 |
| rs1510719 | C | T | 0.383439 | -0.03888 | 0.004947 | 3.84E-15 | 0.0029 | 0.0236 | 0.9017 | 0.000715 | 156.258 |
| rs1592757 | C | G | 0.355772 | 0.031105 | 0.005025 | 6.00E-10 | 0.0162 | 0.0239 | 0.4975 | 0.000444 | 96.91159 |
| rs1596747 | G | A | 0.494136 | 0.031087 | 0.004807 | 1.00E-10 | 0.0177 | 0.0228 | 0.4375 | 0.000483 | 105.5731 |
| rs1716171 | T | C | 0.790024 | 0.038398 | 0.005904 | 7.83E-11 | 0.0326 | 0.0279 | 0.2431 | 0.000489 | 106.8933 |
| rs17379561 | T | A | 0.144391 | 0.053071 | 0.006866 | 1.08E-14 | 0.0259 | 0.0272 | 0.3402 | 0.000696 | 152.1064 |
| rs1883842 | G | T | 0.279255 | 0.030833 | 0.005368 | 9.27E-09 | 0.0521 | 0.0291 | 0.07367 | 0.000383 | 83.61699 |
| rs1937450 | G | T | 0.537739 | 0.031585 | 0.004845 | 7.07E-11 | 0.05 | 0.023 | 0.02932 | 0.000496 | 108.3754 |
| rs2016933 | G | C | 0.730053 | -0.03103 | 0.005421 | 1.04E-08 | -0.0386 | 0.0266 | 0.1472 | 0.000379 | 82.89687 |
| rs2023878 | T | C | 0.192377 | -0.03628 | 0.006119 | 3.04E-09 | -0.0013 | 0.0259 | 0.9586 | 0.000409 | 89.39077 |
| rs2043539 | A | G | 0.41866 | 0.027206 | 0.004865 | 2.24E-08 | 0.041 | 0.0235 | 0.08119 | 0.00036 | 78.71902 |
| rs215614 | A | G | 0.629725 | -0.03285 | 0.004977 | 4.08E-11 | -0.0055 | 0.0253 | 0.8285 | 0.000503 | 109.9972 |
| rs2164300 | T | C | 0.523279 | -0.02648 | 0.004827 | 4.13E-08 | 0.0156 | 0.0228 | 0.4921 | 0.00035 | 76.40701 |
| rs2240326 | A | G | 0.473775 | -0.04717 | 0.004813 | 1.13E-22 | -0.0018 | 0.0228 | 0.9363 | 0.001109 | 242.5666 |
| rs2396133 | G | A | 0.475329 | 0.029355 | 0.004818 | 1.11E-09 | -0.02 | 0.0229 | 0.3816 | 0.00043 | 93.91433 |
| rs2396766 | A | G | 0.47308 | 0.032206 | 0.004819 | 2.34E-11 | -0.0032 | 0.0227 | 0.8875 | 0.000517 | 112.9999 |
| rs2734839 | T | C | 0.606693 | -0.02835 | 0.004928 | 8.79E-09 | -0.0247 | 0.0228 | 0.2778 | 0.000384 | 83.79429 |
| rs2744961 | T | C | 0.358437 | 0.029201 | 0.005015 | 5.81E-09 | 0.0179 | 0.0241 | 0.4588 | 0.000392 | 85.6875 |
| rs2782641 | A | G | 0.612669 | 0.027088 | 0.004946 | 4.33E-08 | 1.00E-04 | 0.0233 | 0.9955 | 0.000348 | 76.09011 |
| rs2815749 | G | A | 0.800974 | 0.038877 | 0.006022 | 1.07E-10 | 0.0075 | 0.0327 | 0.8194 | 0.000482 | 105.2991 |
| rs2834005 | C | T | 0.315 | 0.0297 | 0.005173 | 9.42E-09 | 0.0124 | 0.0254 | 0.6272 | 0.000381 | 83.17237 |
| rs2838771 | C | G | 0.646721 | -0.0281 | 0.005066 | 2.91E-08 | -0.0079 | 0.025 | 0.7515 | 0.000361 | 78.82485 |
| rs324769 | T | C | 0.449179 | -0.02677 | 0.004833 | 3.05E-08 | -0.032 | 0.0233 | 0.1686 | 0.000355 | 77.47935 |
| rs329122 | A | G | 0.419631 | -0.02895 | 0.004884 | 3.05E-09 | -0.0353 | 0.023 | 0.1247 | 0.000408 | 89.2158 |
| rs3766823 | A | G | 0.171467 | 0.03936 | 0.006385 | 7.09E-10 | -0.0032 | 0.0276 | 0.9081 | 0.00044 | 96.18293 |
| rs3793577 | G | A | 0.538279 | 0.027031 | 0.00485 | 2.49E-08 | -0.0021 | 0.0228 | 0.9268 | 0.000363 | 79.355 |
| rs3828917 | T | G | 0.041826 | 0.067111 | 0.012005 | 2.27E-08 | 0.0215 | 0.0912 | 0.8135 | 0.000361 | 78.87644 |
| rs3863241 | T | C | 0.52696 | 0.032498 | 0.004815 | 1.49E-11 | -0.0102 | 0.0231 | 0.6578 | 0.000527 | 115.0618 |
| rs4300861 | T | C | 0.38208 | 0.030713 | 0.004949 | 5.43E-10 | 0.0129 | 0.023 | 0.5743 | 0.000445 | 97.32819 |
| rs4382592 | G | T | 0.699524 | -0.03027 | 0.005251 | 8.20E-09 | -0.0338 | 0.0254 | 0.1846 | 0.000385 | 84.14974 |
| rs4713692 | T | C | 0.367808 | -0.02761 | 0.004986 | 3.07E-08 | -0.0288 | 0.0239 | 0.2295 | 0.000355 | 77.47357 |
| rs569356 | G | A | 0.140835 | -0.03792 | 0.00691 | 4.07E-08 | 0.0026 | 0.0337 | 0.9396 | 0.000348 | 76.02572 |
| rs6711584 | A | G | 0.452019 | 0.032255 | 0.00484 | 2.66E-11 | 0.0186 | 0.0228 | 0.414 | 0.000515 | 112.6251 |
| rs6722661 | A | G | 0.364669 | -0.03225 | 0.005004 | 1.15E-10 | 0.0513 | 0.0238 | 0.03106 | 0.000482 | 105.3397 |
| rs6780459 | T | A | 0.746622 | 0.030551 | 0.005521 | 3.14E-08 | -0.0169 | 0.0278 | 0.542099 | 0.000353 | 77.15674 |
| rs7032155 | A | C | 0.59185 | 0.02775 | 0.004914 | 1.63E-08 | 0.0593 | 0.0235 | 0.01163 | 0.000372 | 81.2882 |
| rs7206608 | G | C | 0.322927 | 0.029154 | 0.005145 | 1.46E-08 | 0.0238 | 0.0237 | 0.3151 | 0.000372 | 81.20992 |
| rs7241572 | A | G | 0.209101 | 0.036551 | 0.005975 | 9.49E-10 | 0.0045 | 0.0278 | 0.8701 | 0.000442 | 96.55582 |
| rs7527682 | G | A | 0.53725 | -0.02668 | 0.004822 | 3.13E-08 | -0.0371 | 0.0234 | 0.1134 | 0.000354 | 77.35475 |
| rs7541875 | G | A | 0.426069 | 0.027397 | 0.00485 | 1.61E-08 | -0.021 | 0.0233 | 0.366 | 0.000367 | 80.20841 |
| rs7600261 | T | C | 0.306391 | 0.033803 | 0.005221 | 9.47E-11 | -0.0148 | 0.0247 | 0.549201 | 0.000486 | 106.1282 |
| rs7612999 | A | G | 0.245338 | 0.030523 | 0.005595 | 4.90E-08 | 0.0333 | 0.0269 | 0.2155 | 0.000345 | 75.37597 |
| rs761777 | G | A | 0.254034 | 0.034534 | 0.005545 | 4.71E-10 | -0.013 | 0.0255 | 0.6092 | 0.000452 | 98.76703 |
| rs7675588 | A | C | 0.794635 | -0.03352 | 0.005954 | 1.80E-08 | -0.0058 | 0.0306 | 0.8486 | 0.000367 | 80.13874 |
| rs7685686 | G | A | 0.422353 | -0.02792 | 0.004892 | 1.14E-08 | -0.0237 | 0.0229 | 0.3003 | 0.00038 | 83.12107 |
| rs773109 | A | G | 0.335269 | -0.03806 | 0.005102 | 8.71E-14 | -0.0072 | 0.0247 | 0.769899 | 0.000646 | 141.0919 |
| rs7942368 | T | C | 0.214659 | -0.03397 | 0.005919 | 9.54E-09 | 0.0346 | 0.0279 | 0.2153 | 0.000389 | 85.00265 |
| rs903678 | A | G | 0.339412 | 0.027738 | 0.005085 | 4.89E-08 | 0.0161 | 0.0249 | 0.5175 | 0.000345 | 75.38071 |
| rs903959 | A | T | 0.399262 | 0.029163 | 0.004916 | 2.99E-09 | -0.0315 | 0.0234 | 0.1777 | 0.000408 | 89.14483 |
| rs9372625 | A | G | 0.383042 | -0.03773 | 0.004954 | 2.62E-14 | -0.0148 | 0.0244 | 0.5446 | 0.000673 | 147.0304 |
| rs9373363 | G | A | 0.253631 | -0.03268 | 0.00556 | 4.13E-09 | -0.0133 | 0.0242 | 0.580801 | 0.000404 | 88.36898 |
| rs9396740 | A | G | 0.248794 | -0.03149 | 0.005559 | 1.47E-08 | -0.0083 | 0.0282 | 0.7671 | 0.000371 | 81.00215 |
| rs942065 | A | G | 0.634045 | 0.030738 | 0.005009 | 8.45E-10 | -0.0026 | 0.0235 | 0.911 | 0.000438 | 95.80964 |
| rs9529055 | A | G | 0.475633 | 0.02666 | 0.004816 | 3.11E-08 | 0.0473 | 0.0228 | 0.03777 | 0.000355 | 77.46457 |
| rs9542729 | G | C | 0.20244 | -0.03632 | 0.005999 | 1.41E-09 | -0.0107 | 0.0255 | 0.6745 | 0.000426 | 93.07446 |
| rs9615905 | T | C | 0.458193 | 0.027566 | 0.004838 | 1.21E-08 | 0.002 | 0.0229 | 0.9289 | 0.000377 | 82.43345 |
| rs9636202 | A | G | 0.26663 | -0.03504 | 0.005472 | 1.51E-10 | -0.0189 | 0.0273 | 0.4894 | 0.00048 | 104.9486 |
| rs9940128 | A | G | 0.421755 | 0.033251 | 0.004863 | 8.06E-12 | 0.0577 | 0.0229 | 0.01173 | 0.000539 | 117.8507 |

**Table S2** The allele frequencies in an average European population

|  | Ref Allele | Alt Allele |
| --- | --- | --- |
| rs1011407 | A=0.88498 | G=0.11502 |
| rs10133111 | G=0.833213 | A=0.166787 |
| rs1021363 | A=0.35100 | G=0.64900 |
| rs10837002 | C=0.66569 | G=0.33431 |
| rs11762636 | C=0.86149 | A=0.13851, T=0.00000 |
| rs11953061 | C=0.65274 | T=0.34726 |
| rs12204714 | C=0.33991 | T=0.66009 |
| rs12357321 | G=0.67805 | A=0.32195 |
| rs12453010 | C=0.60297 | T=0.39703 |
| rs12598916 | C=0.73037 | G=0.26963 |
| rs12997558 | G=0.645906 | A=0.354094 |
| rs1334297 | G=0.26321 | A=0.73679 |
| rs13409451 | A=0.63468 | G=0.36532 |
| rs1431196 | A=0.57297 | G=0.42703 |
| rs1479405 | C=0.67829 | T=0.32171 |
| rs1592757 | G=0.64672 | C=0.35328 |
| rs1596747 | A=0.51629 | G=0.48371, T=0.00000 |
| rs1716171 | C=0.21366 | T=0.78634 |
| rs17379561 | A=0.90967 | G=0.00000, T=0.09033 |
| rs1883842 | T=0.82850 | A=0.00000, C=0.00000, G=0.17150 |
| rs1937450 | T=0.46724 | G=0.53276 |
| rs2016933 | C=0.29707 | G=0.70293 |
| rs2023878 | C=0.818499 | T=0.181501 |
| rs2043539 | G=0.58414 | A=0.41586 |
| rs2106353 | G=0.80138 | T=0.19862 |
| rs2164300 | C=0.509854 | T=0.490146 |
| rs2240326 | G=0.501520 | A=0.498480 |
| rs2396133 | A=0.54452 | G=0.45548 |
| rs2396766 | G=0.55018 | A=0.44982 |
| rs2734839 | C=0.5164 | A=0.0000, T=0.4836 |
| rs2834005 | T=0.71555 | C=0.28445, G=0.00000 |
| rs2838771 | G=0.4171 | C=0.5829, T=0.0000 |
| rs324769 | C=0.59113 | A=0.00000, T=0.40887 |
| rs3766823 | G=0.829546 | A=0.170454 |
| rs3793577 | A=0.477852 | C=0.000000, G=0.522148 |
| rs3828917 | G=0.97450 | A=0.00000, T=0.02550 |
| rs3863241 | C=0.47081 | A=0.00000, T=0.52919 |
| rs4300861 | C=0.61802 | T=0.38198 |
| rs4382592 | T=0.30319 | G=0.69681 |
| rs4713692 | C=0.6524 | T=0.3476 |
| rs569356 | A=0.866750 | G=0.133250 |
| rs6722661 | G=0.64746 | A=0.35254 |
| rs6780459 | A=0.23492 | T=0.76508 |
| rs7032155 | C=0.38658 | A=0.61342 |
| rs7206608 | C=0.77160 | A=0.00000, G=0.22840 |
| rs7241572 | G=0.80113 | A=0.19887 |
| rs7527682 | A=0.464289 | C=0.000000, G=0.535711 |
| rs7541875 | A=0.60207 | G=0.39793 |
| rs7600261 | C=0.78798 | G=0.00000, T=0.21202 |
| rs7612999 | G=0.73775 | A=0.26225 |
| rs761777 | A=0.73905 | G=0.26095 |
| rs7675588 | C=0.2437 | A=0.7563, T=0.0000 |
| rs7685686 | A=0.581261 | G=0.418739 |
| rs773109 | G=0.68232 | A=0.31768 |
| rs7942368 | C=0.762285 | T=0.237715 |
| rs903959 | T=0.60137 | A=0.39863 |
| rs9372625 | G=0.619918 | A=0.380082 |
| rs9373363 | A=0.762576 | C=0.000000, G=0.237424 |
| rs9396740 | G=0.728513 | A=0.271487 |
| rs942065 | G=0.35622 | A=0.64378 |
| rs9517313 | G=0.63692 | C=0.36308 |
| rs9529055 | G=0.56748 | A=0.43252, C=0.00000, T=0.00000 |
| rs9542729 | C=0.86748 | A=0.00000, G=0.13252 |
| rs9615905 | C=0.565841 | A=0.000000, G=0.000000, T=0.434159 |
| rs9636202 | G=0.77165 | A=0.22835, C=0.00000 |

**Figure S1** MR Scatter plot for the genetic association between GERD and risk of COPD

**
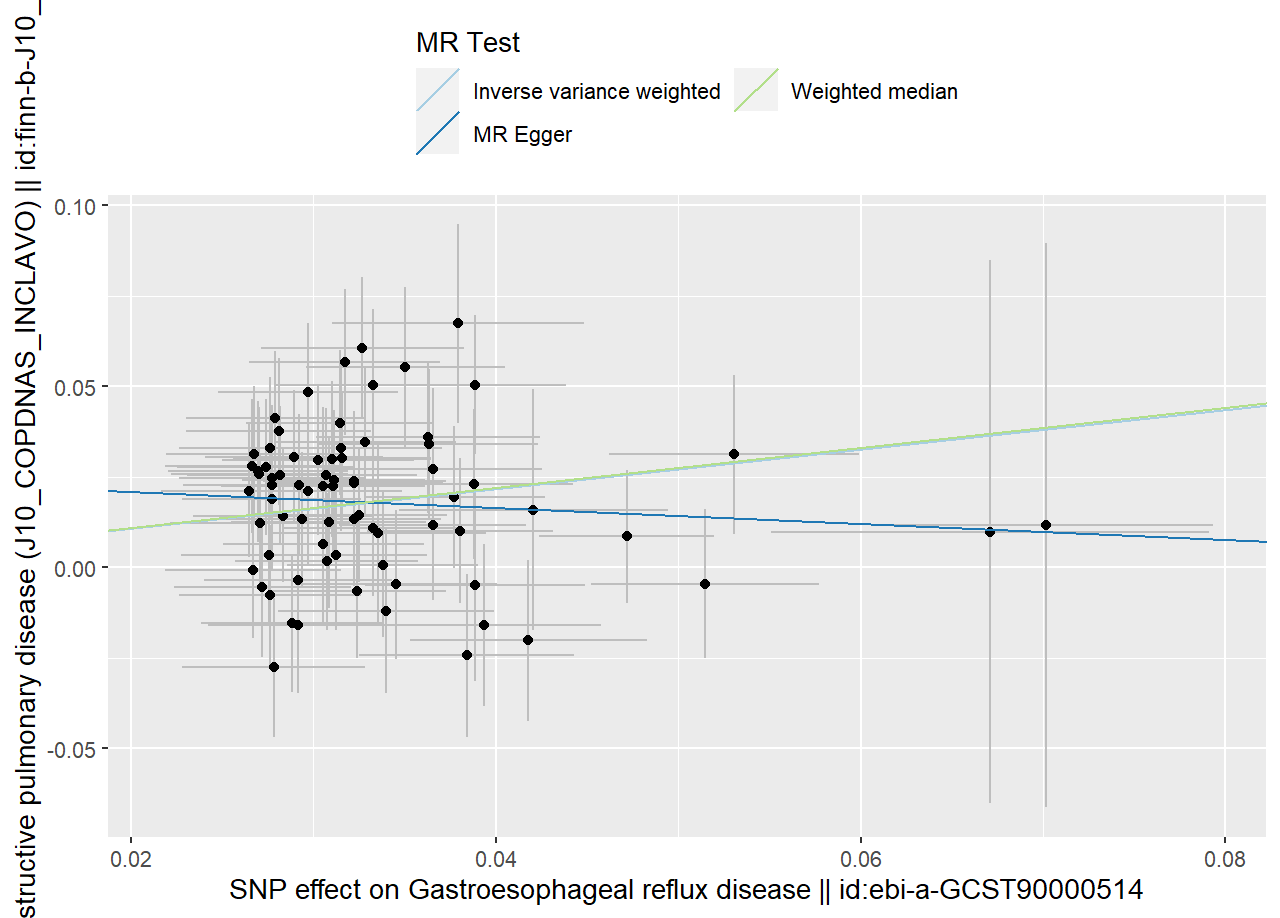
**

**Figure S2** MR Scatter plot for the genetic association between GERD and risk of bronchitis


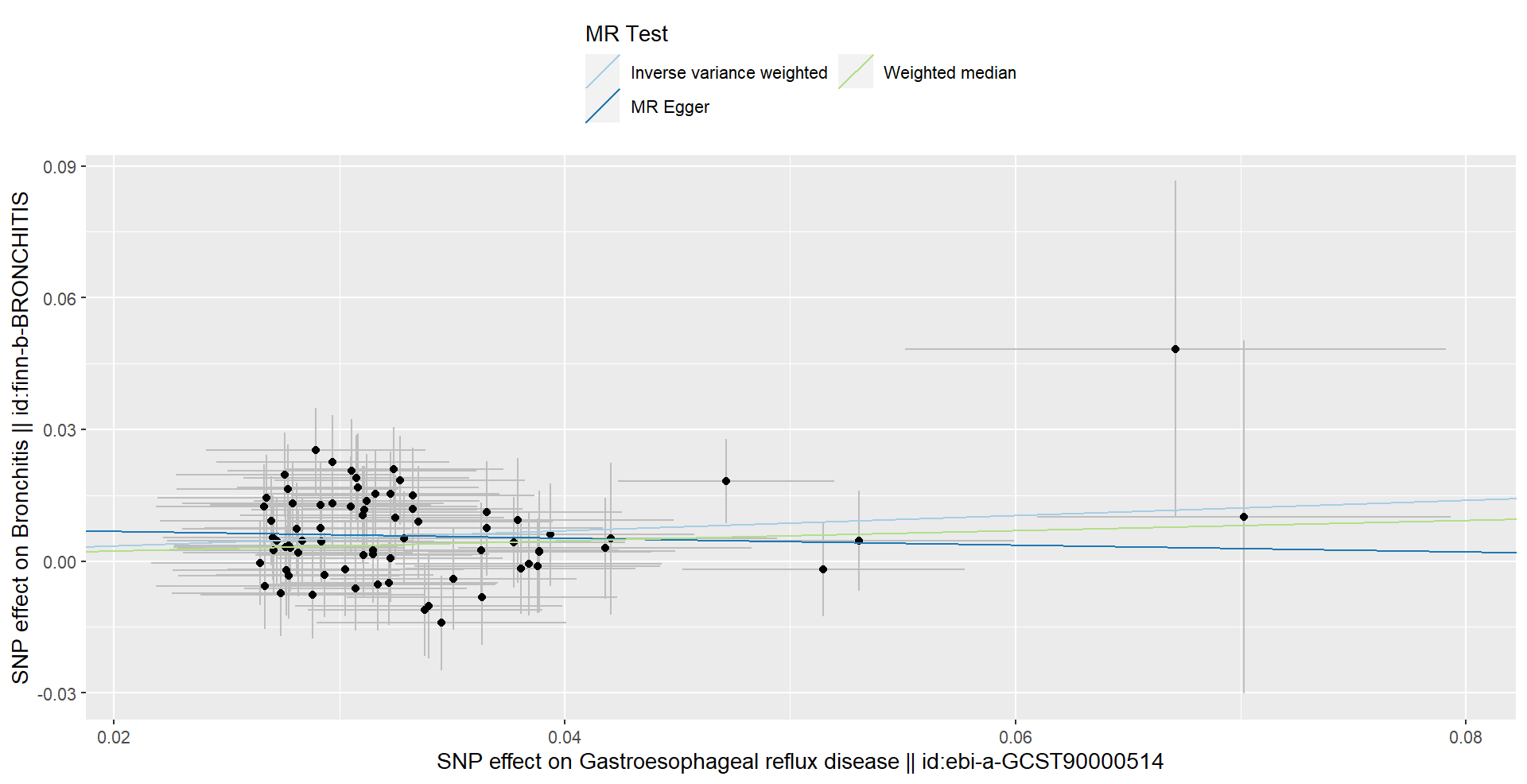


**Figure S3** MR Scatter plot for the genetic association between GERD and risk of pneumonia


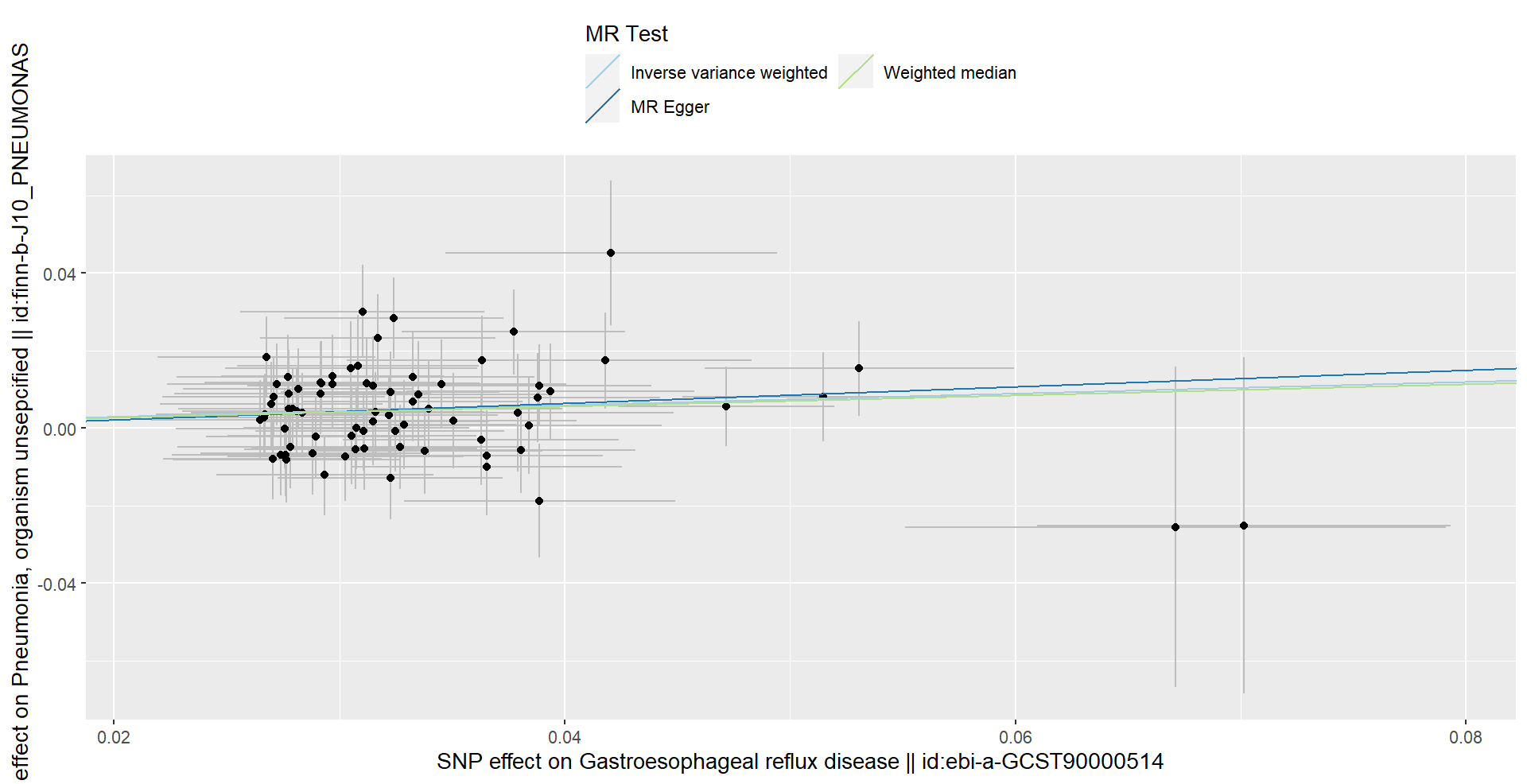


**Figure S4** MR Scatter plot for the genetic association between GERD and risk of lung cancer


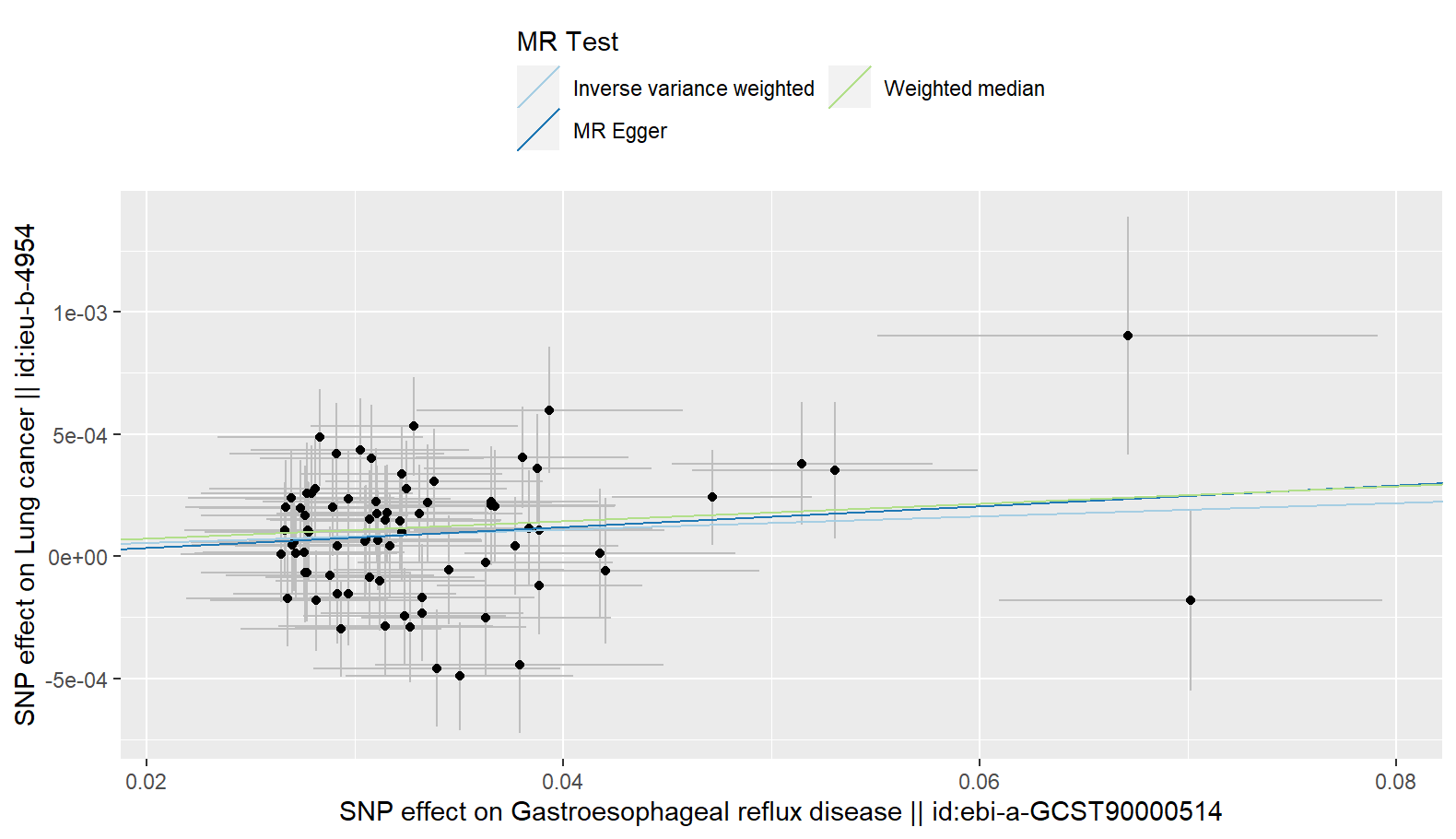


**Figure S5** MR Scatter plot for the genetic association between GERD and risk of pulmonary embolism


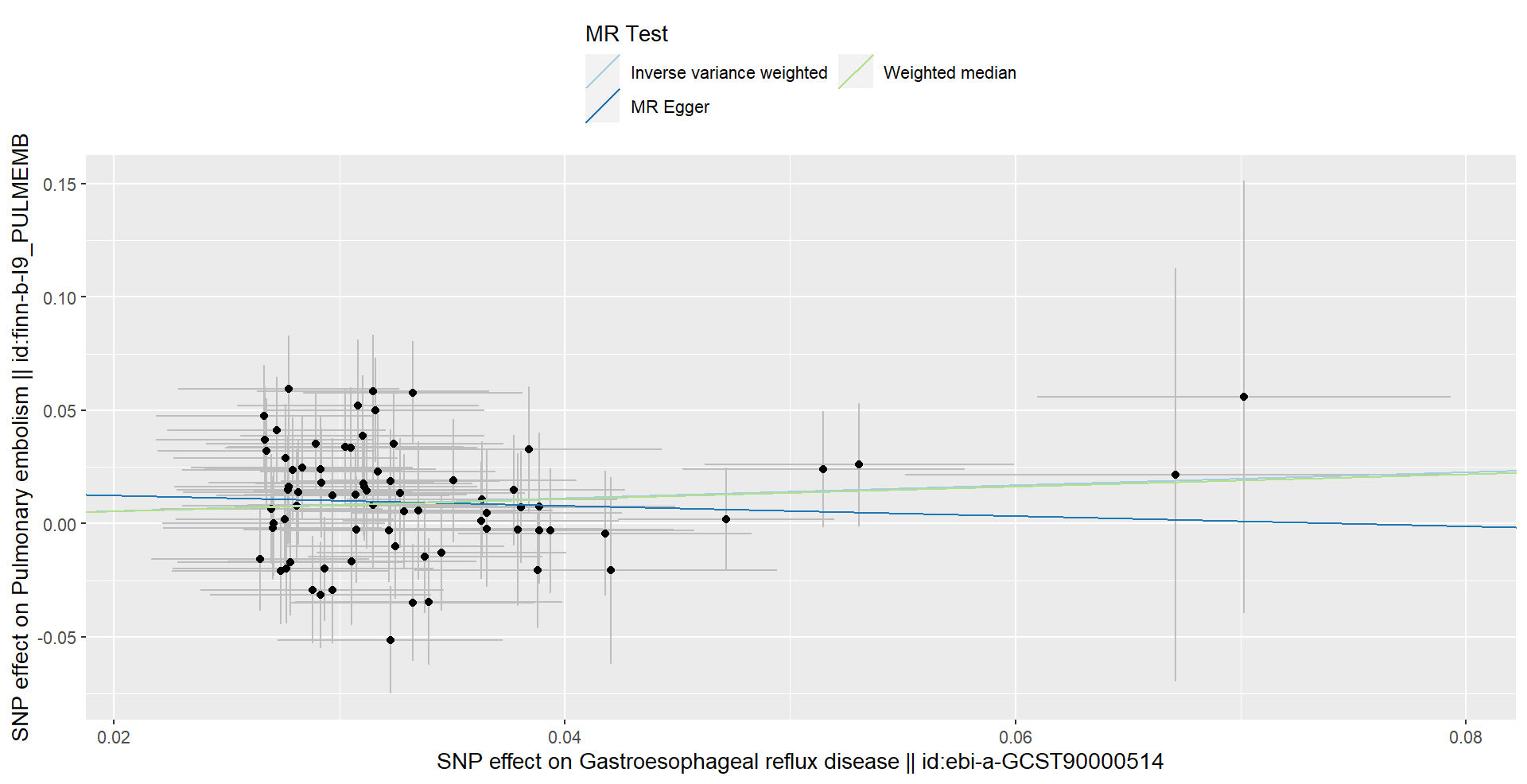


**Figure S6** MR Funnel plot for the genetic association between GERD and risk of COPD


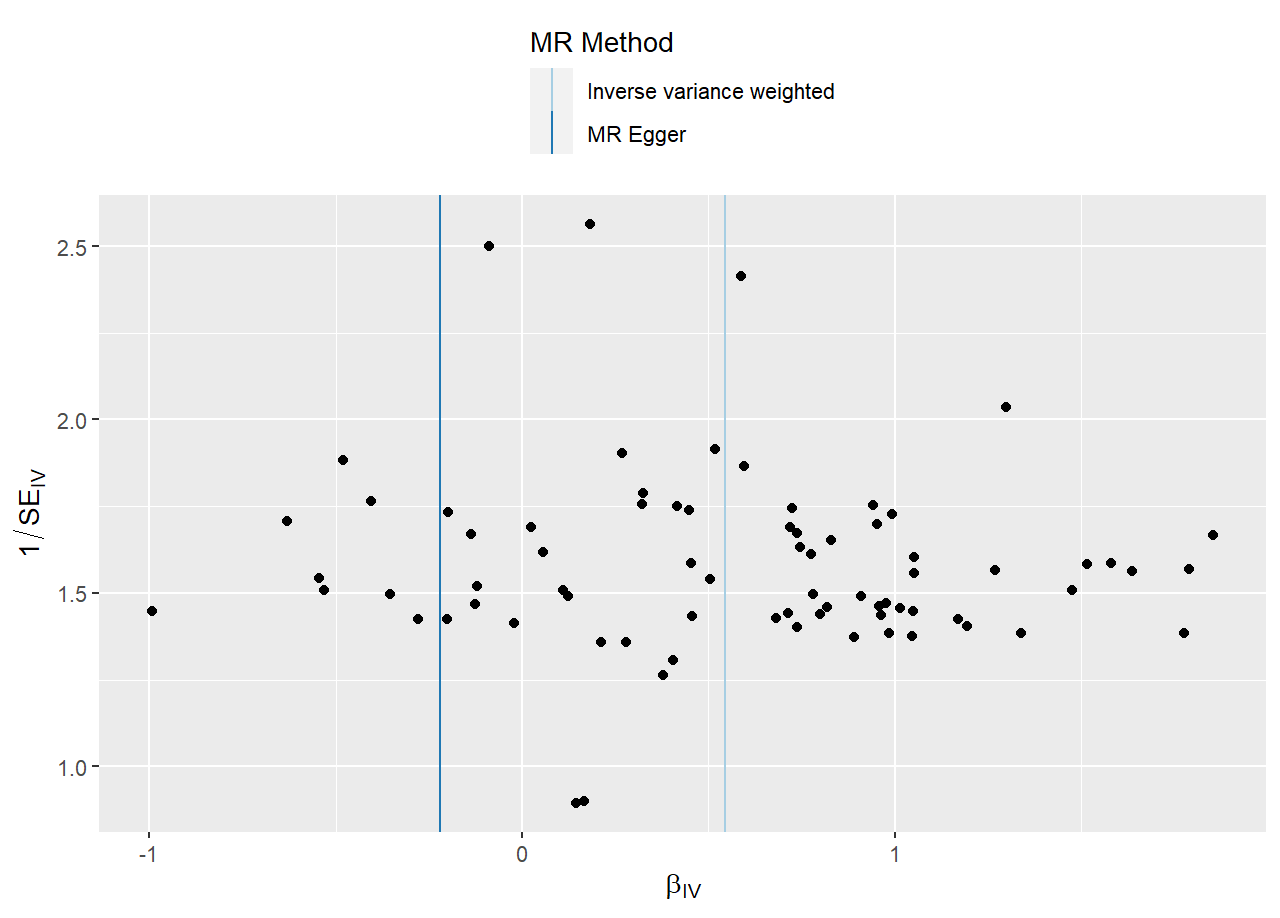


**Figure S7** MR Funnel plot for the genetic association between GERD and risk of bronchitis


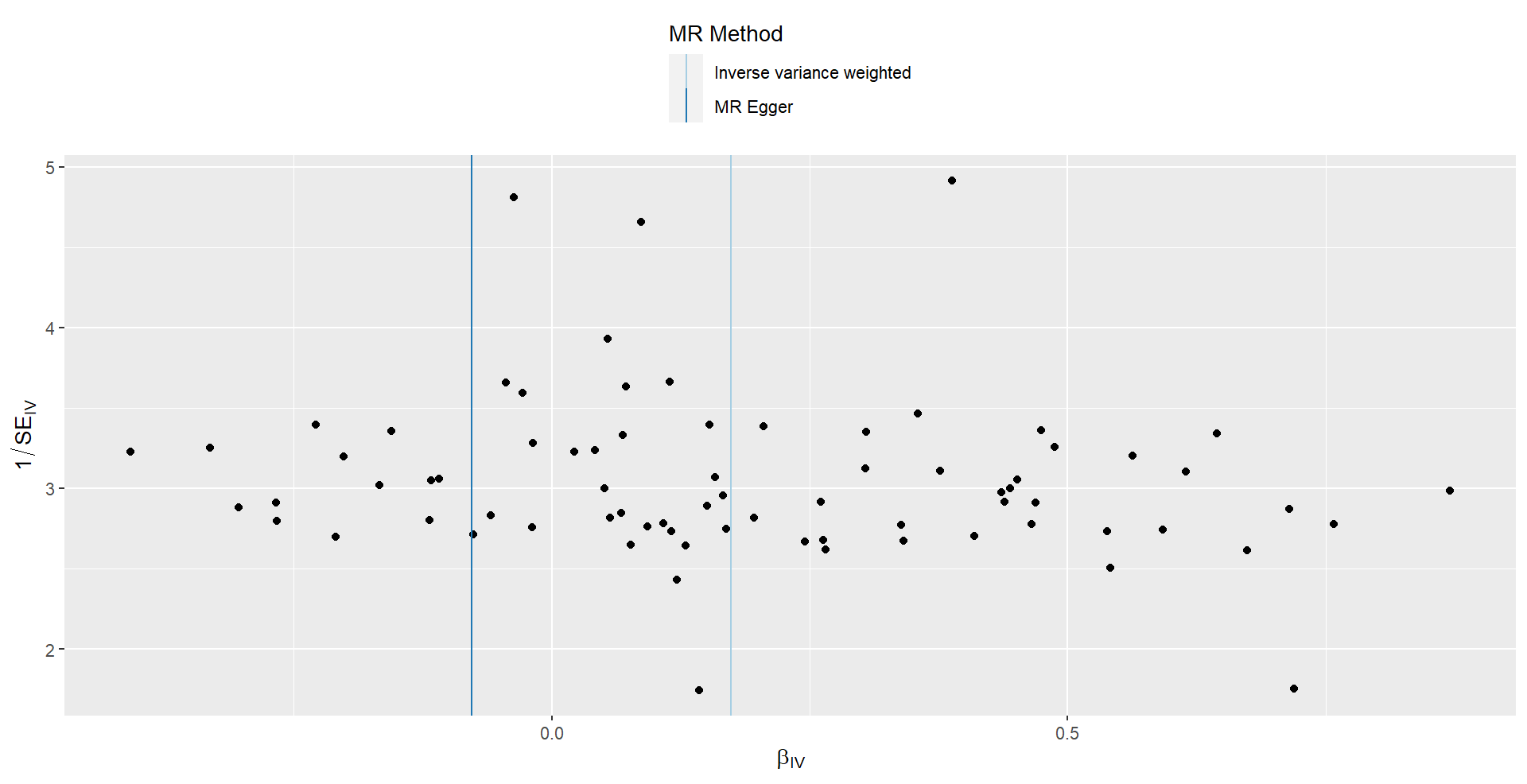


**Figure S8** MR Funnel plot for the genetic association between GERD and risk of pneumonia


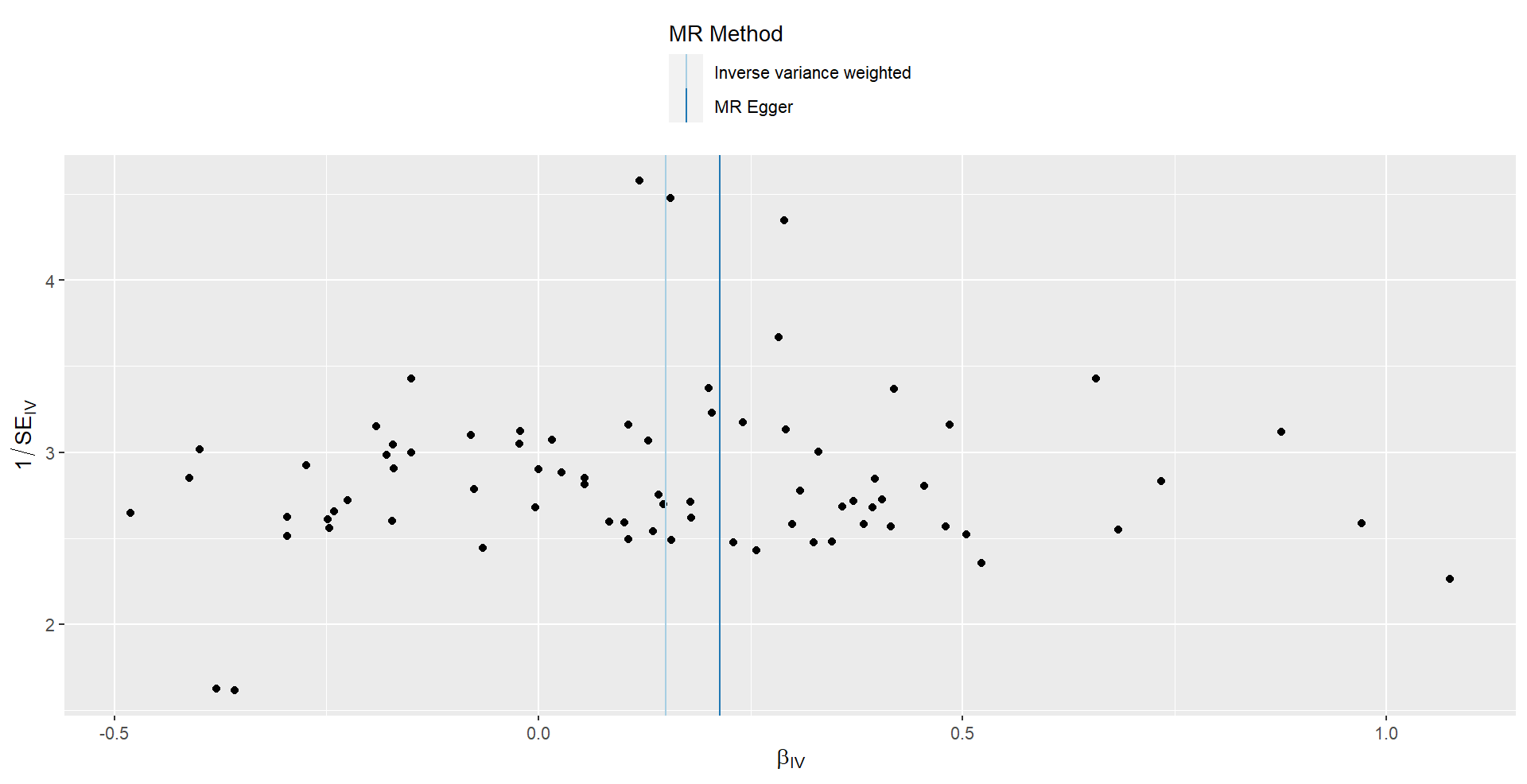


**Figure S9** MR Funnel plot for the genetic association between GERD and risk of lung cancer


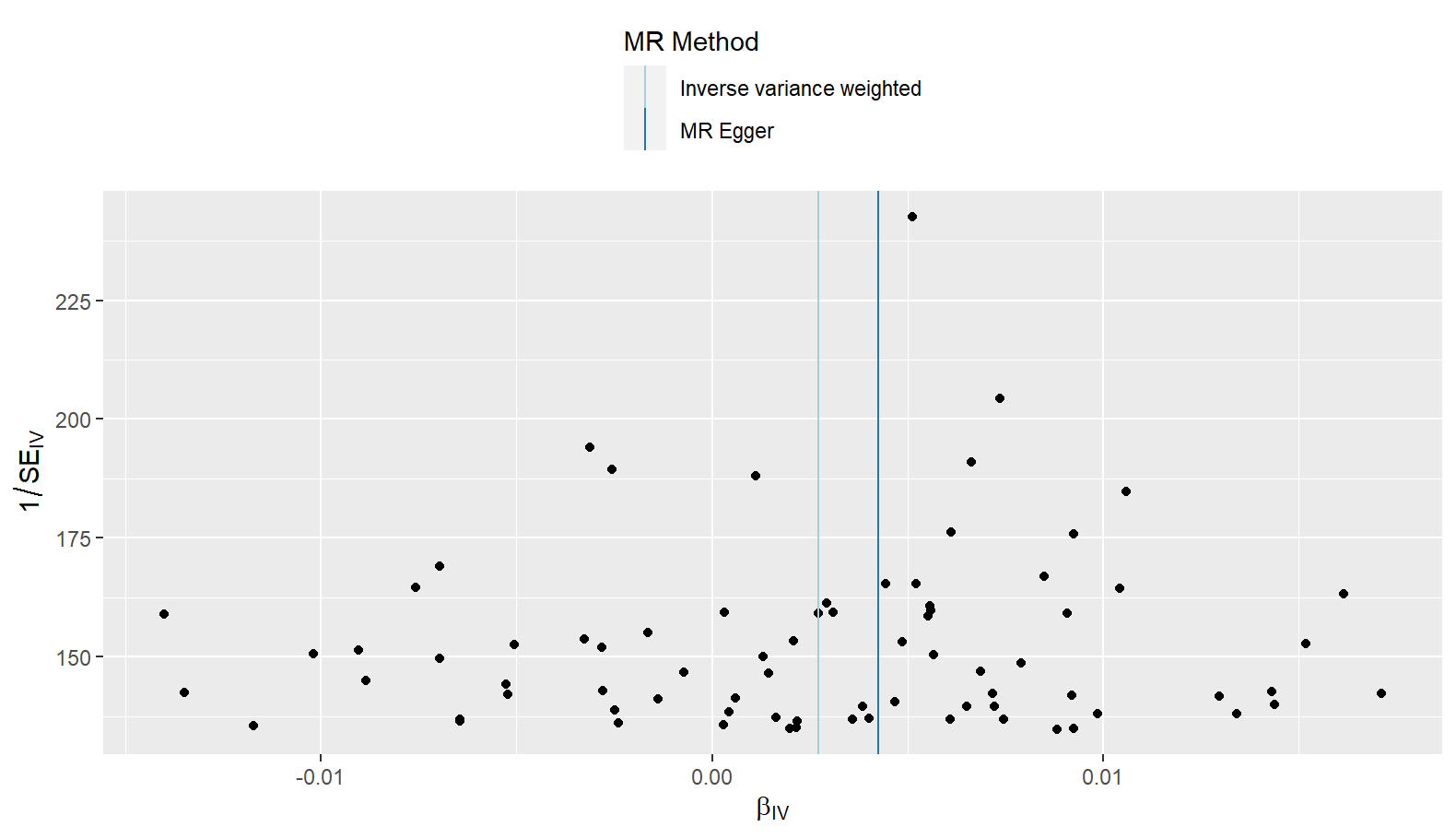


**Figure S10** MR Funnel plot for the genetic association between GERD and risk of pulmonary embolism


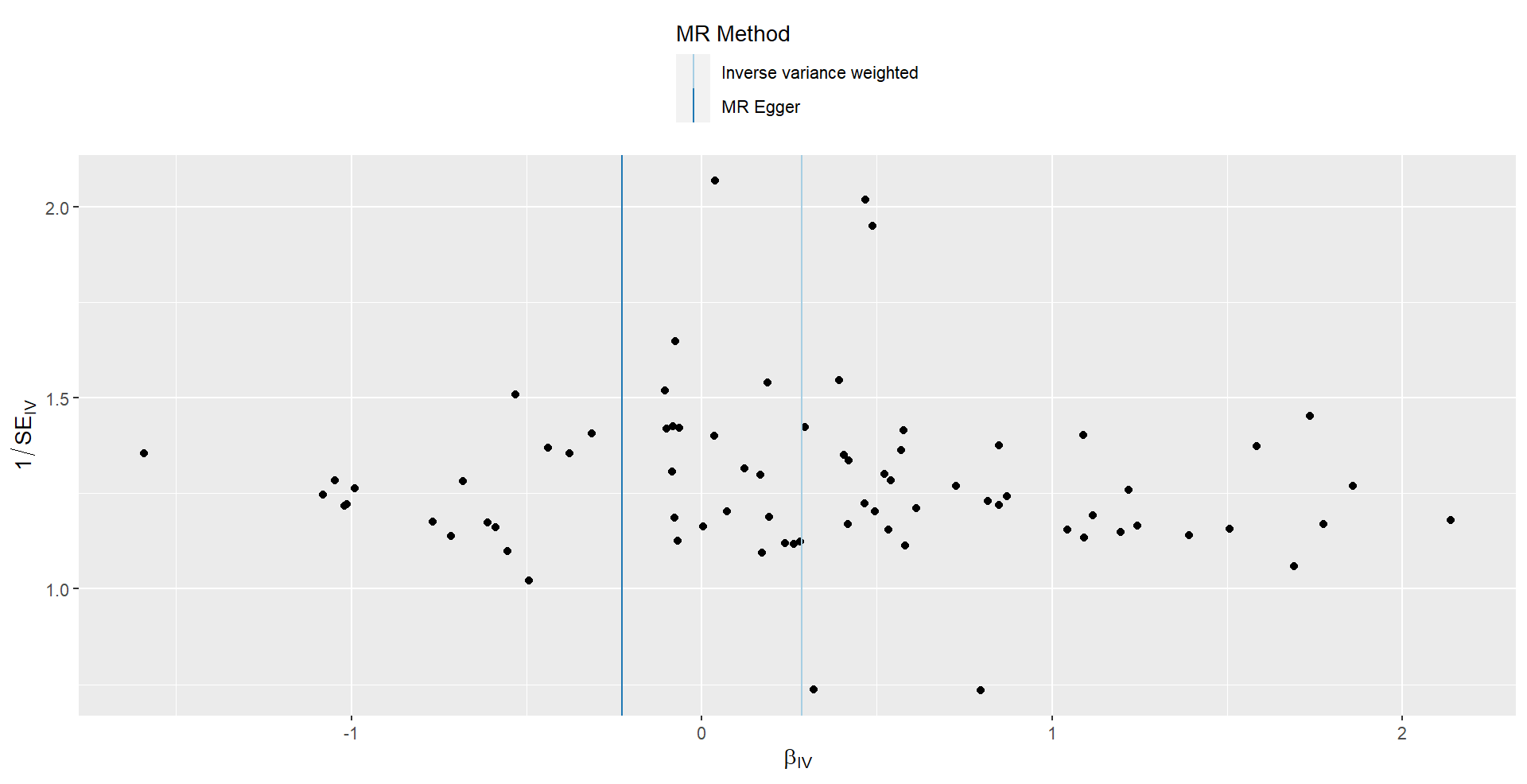


**Figure S11** MR Leave-one-SNP-out plot for the genetic association between GERD and risk of COPD


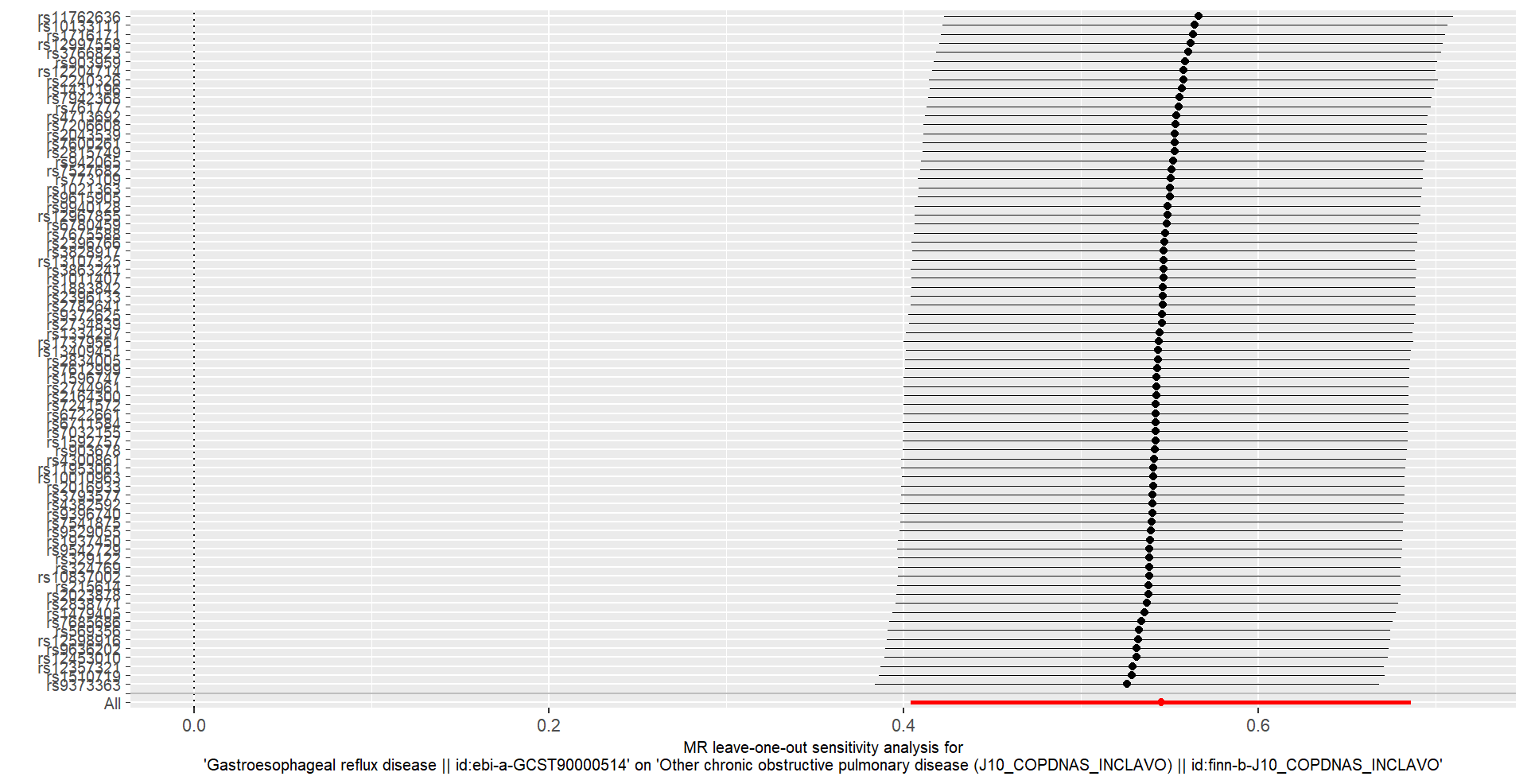


**Figure S12** MR Leave-one-SNP-out plot for the genetic association between GERD and risk of bronchitis


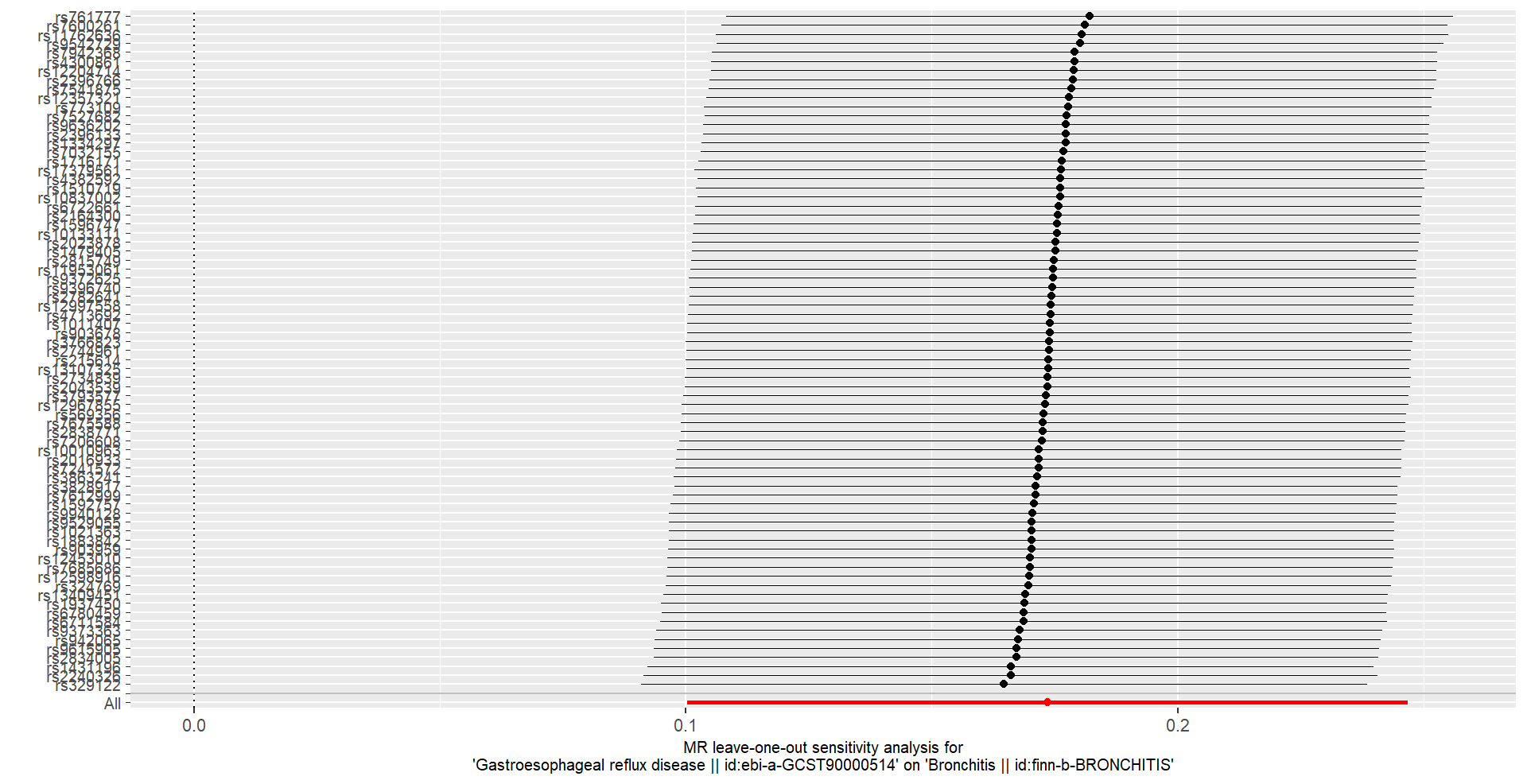


**Figure S13** MR Leave-one-SNP-out plot for the genetic association between GERD and risk of pneumonia


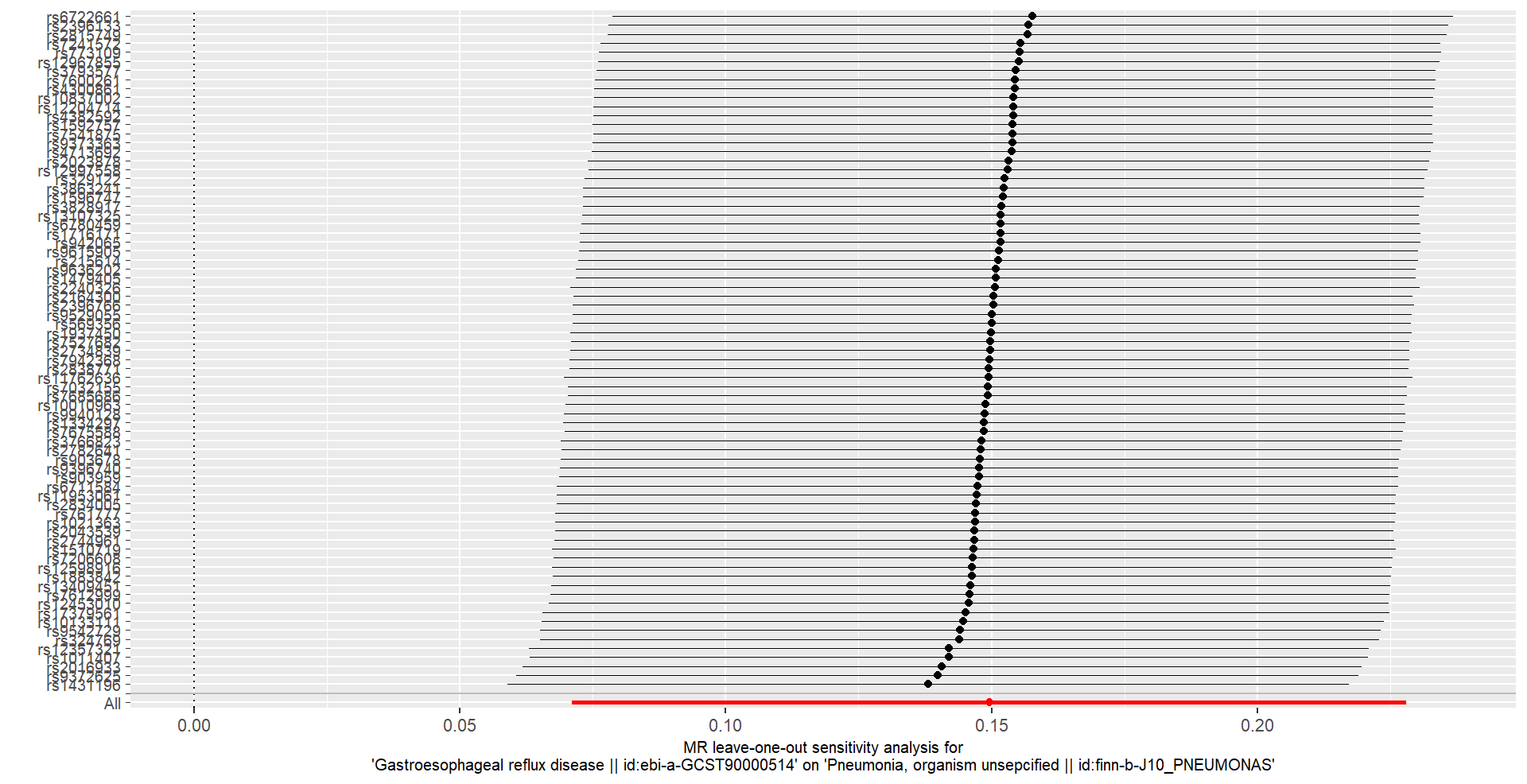


**Figure S14** MR Leave-one-SNP-out plot for the genetic association between GERD and risk of lung cancer


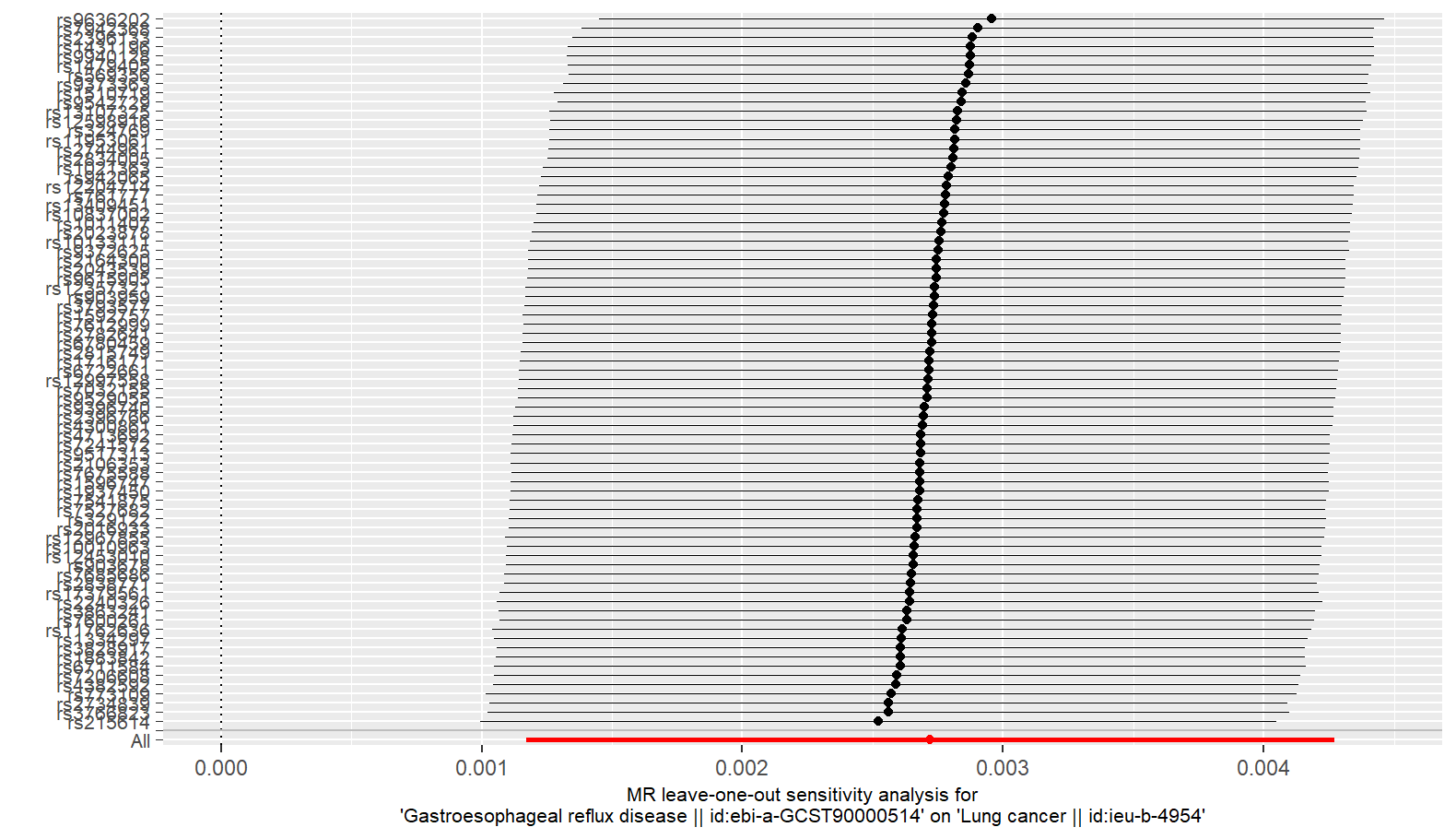


**Figure S15** MR Leave-one-SNP-out plot for the genetic association between GERD and risk of pulmonary embolism


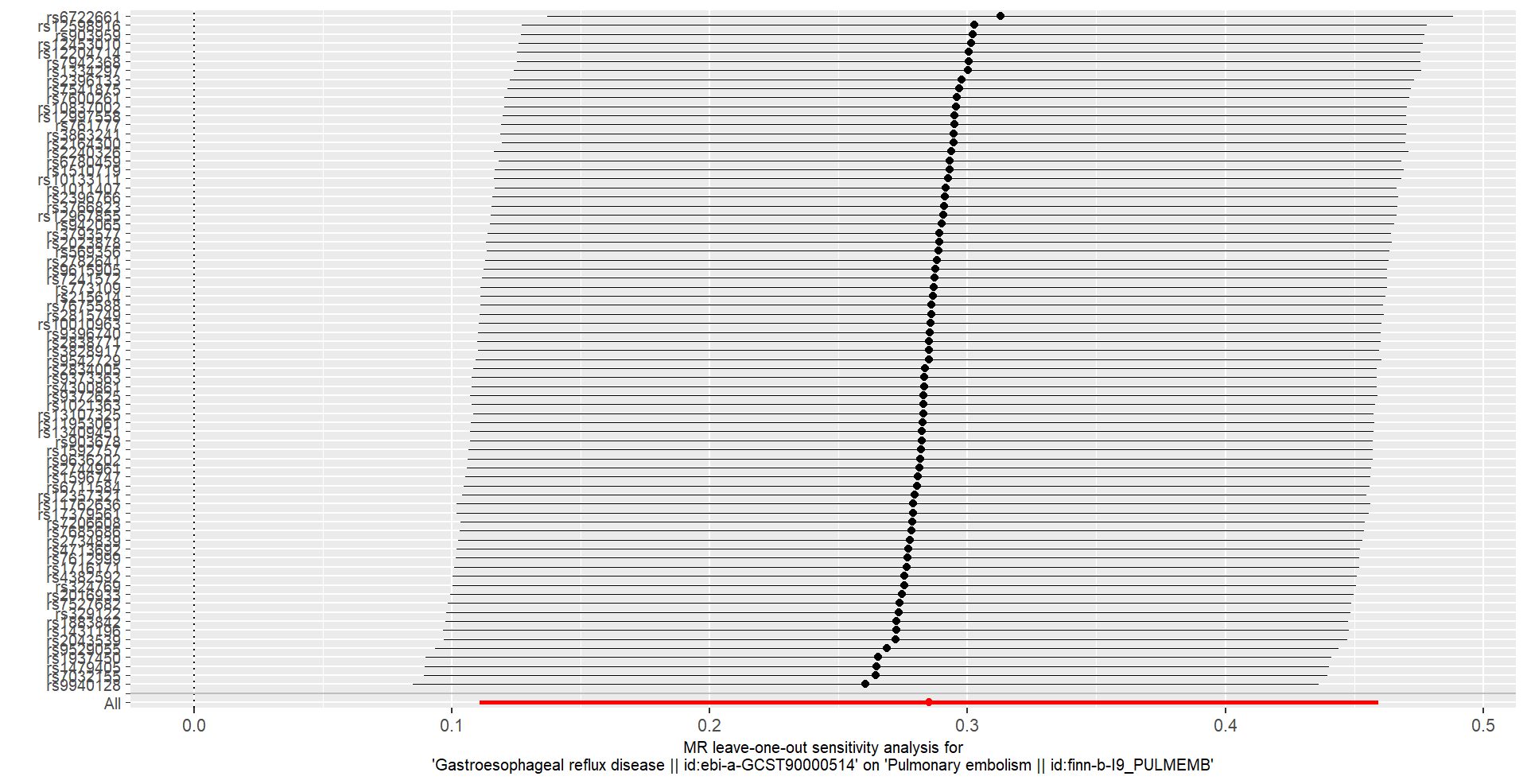

Supplement: Supplementary file 1 — Additional file 1: Table S1. Genetic variants used in the analyses investigating a causal impact of genetically predicted GERD and respiratory diseases. Table S2. The allele frequencies in an average European population. Figure S1. MR Scatter plot for the genetic association between GERD and risk of COPD. Figure S2. MR Scatter plot for the genetic association between GERD and risk of bronchitis. Figure S3. MR Scatter plot for the genetic association between GERD and risk of pneumonia. Figure S4. MR Scatter plot for the genetic association between GERD and risk of lung cancer. Figure S5. MR Scatter plot for the genetic association between GERD and risk of pulmonary embolism. Figure S6. MR Funnel plot for the genetic association between GERD and risk of COPD. Figure S7. MR Funnel plot for the genetic association between GERD and risk of bronchitis. Figure S8. MR Funnel plot for the genetic association between GERD and risk of pneumonia. Figure S9. MR Funnel plot for the genetic association between GERD and risk of lung cancer. Figure S10. MR Funnel plot for the genetic association between GERD and risk of pulmonary embolism. Figure S11. MR Leave-one-SNP-out plot for the genetic association between GERD and risk of COPD. Figure S12. MR Leave-one-SNP-out plot for the genetic association between GERD and risk of bronchitis. Figure S13. MR Leave-one-SNP-out plot for the genetic association between GERD and risk of pneumonia. Figure S14. MR Leave-one-SNP-out plot for the genetic association between GERD and risk of lung cancer. Figure S15. MR Leave-one-SNP-out plot for the genetic association between GERD and risk of pulmonary embolism. [file 12967_2023_4786_MOESM1_ESM.docx]
