# Supplementary figures and images for: Gastroesophageal reflux disease and the risk of respiratory diseases: a Mendelian randomization study
Source: J Transl Med. 2024 Jan 16;22:60. doi: 10.1186/s12967-023-04786-0 (PMC10790464; doi:10.1186/s12967-023-04786-0)

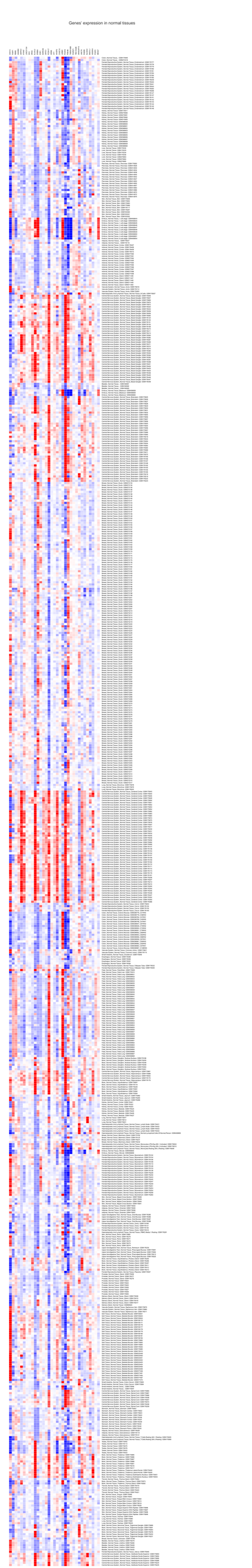

Supplement: Supplementary file 2 — Additional file 2. Genes’ expression in normal tissues. [file 12967_2023_4786_MOESM2_ESM.pdf]
